# Global Occurrence of Cyanotoxins in Drinking Water Systems: Recent Advances, Human Health Risks, Mitigation, and Future Directions

**DOI:** 10.3390/life15050825

**Published:** 2025-05-21

**Authors:** Jerikias Marumure, Willis Gwenzi, Zakio Makuvara, Tinoziva T. Simbanegavi, Richwell Alufasi, Marvelous Goredema, Claudious Gufe, Rangarirayi Karidzagundi, Piotr Rzymski, Dariusz Halabowski

**Affiliations:** 1 Department of Physics, Geography and Environmental Science, School of Natural Sciences, Great Zimbabwe University, Masvingo, Zimbabwe; jmarumure@gzu.ac.zw (J.M.); zmakuvara@gzu.ac.zw (Z.M.); 2 Department of Life and Consumer Sciences, School of Agriculture and Life Sciences, College of Agriculture and Environmental Sciences, University of South Africa, Pretoria 0002, South Africa; 3 Formerly Alexander von Humboldt Fellow, Leibniz-Institut für Agrartechnik und Bioökonomie e.V. (ATB), Max-Eyth-Allee 100, D-14469 Potsdam, Germany; wgwenzi@yahoo.co.uk; 4 Formerly Alexander von Humboldt Fellow, Grassland Science and Renewable Plant Resources, Faculty of Organic Agricultural Sciences, Universität Kassel, Steinstraße 19, D-37213 Witzenhausen, Germany; 5 Independent Researcher, Biosystems & Environmental Engineering Research Group, 380 New Adylin, Westgate, Harare, Zimbabwe; 6 Department of Soil Science and Environment, Faculty of Agriculture, Environment, and Food Systems, University of Zimbabwe, P.O. Box MP 167, Mount Pleasant, Harare, Zimbabwe; tinozivasimbanegavi@gmail.com; 7 Biological Sciences Department, Bindura University of Science Education, 741 Chimurenga Road, Off Trojan Road, Bindura, Zimbabwe; alufasirichie@gmail.com (R.A.); marvelousgoredema@gmail.com (M.G.); 8 Department of Veterinary Technical Services, Central Veterinary Laboratories, P.O. Box CY55, 18A Borrowdale Road, Harare, Zimbabwe; claudiousgufe3@gmail.com; 9 Materials Development Unit, Zimbabwe Open University, P.O. Box MP1119, Mount Pleasant, Harare, Zimbabwe; krangarirayi@gmail.com; 10 Department of Environmental Medicine, Poznan University of Medical Sciences, 60-806 Poznań, Poland; rzymskipiotr@ump.edu.pl; 11 University of Lodz, Faculty of Biology and Environmental Protection, Department of Ecology and Vertebrate Zoology, 90-237 Lodz, Poland

**Keywords:** harmful algal blooms, cyanotoxins, drinking water, microcystins, public health

## Abstract

This paper applies a semi-quantitative approach to review the diversity, environmental controls, detection methods, human health risks, and mitigation of cyanotoxins in drinking water systems (DWSs). It discusses the environmental factors controlling the occurrence of cyanotoxins, presents the merits and limitations of emerging methods of their detection (qPCR, liquid chromatography–mass spectrometry, and electrochemical biosensors), and outlines the human exposure pathways and health outcomes with identification of high-risk groups and settings. High-risk groups include (1) communities relying on untreated drinking water from unsafe, polluted water sources and (2) low-income countries where cyanotoxins are not routinely monitored in DWSs. The fate and behavior processes are discussed, including removing cyanotoxins in DWSs based on conventional and advanced treatment processes. The available methods for cyanotoxin removal presented in this paper include (1) polymer-based adsorbents, (2) coagulation/flocculation, (3) advanced oxidation processes, (4) ultra- and nanofiltration, and (5) multi-soil layer systems. Future research should address (1) detection and fate in storage and conveyance facilities and at the point of consumption, (2) degradation pathways and toxicity of by-products or metabolites, (3) interactive health effects of cyanotoxins with legacy and emerging contaminants, (4) removal by low-cost treatment techniques (e.g., solar disinfection, boiling, bio-sand filtration, and chlorination), (5) quantitative health risk profiling of high-risk groups, and (6) epidemiological studies to link the prevalence of human health outcomes (e.g., cancer) to cyanotoxins in DWSs.

## 1. Introduction

Cyanobacterial Harmful Algal Blooms (CyanoHABs) occur in diverse surface waters, including those serving as drinking water sources [1]. These blooms are characterized by the rapid proliferation of Cyanobacteria, some of which produce toxic metabolites known as cyanotoxins [2]. They constitute a diverse group of chemical compounds that differ in mechanisms of toxic action.

The occurrence of cyanotoxins in different water systems has been extensively studied in high-income countries [3,4,5,6] and, to a lesser extent, in developing countries [7,8,9]. However, research remains limited in low-income regions like Africa, where studies have been conducted, e.g., in Zimbabwe [10,11], Ethiopia [12,13], Kenya [14,15], and Uganda [16,17,18] (Table S1). Some studies have also investigated and reported harmful algae and cyanotoxins in sources of drinking water [11,17], drinking water treatment facilities [19], containerized drinking water in households [20], and mineral water [21]. Cyanotoxins in drinking water pose risks to human health, primarily through ingestion.

Recent reviews have explored various aspects of cyanotoxins, including (1) their presence in aquatic ecosystems and organisms such as fish, with a limited focus on drinking water systems [9,22], (2) the diversity of Cyanobacteria and cyanotoxins [7,23], and (3) methods for removing cyanotoxins from drinking water [24,25,26]. However, a comprehensive review of the occurrence of cyanotoxins in drinking water systems (DWSs), their detection, exposure risks, health hazards, and mitigation strategies is currently lacking. DWSs include the entire continuum from source water (surface and groundwater) to water treatment, storage, distribution, and the point of use (e.g., tap water, containerized drinking water, and bottled mineral water).

**Figure 1:**
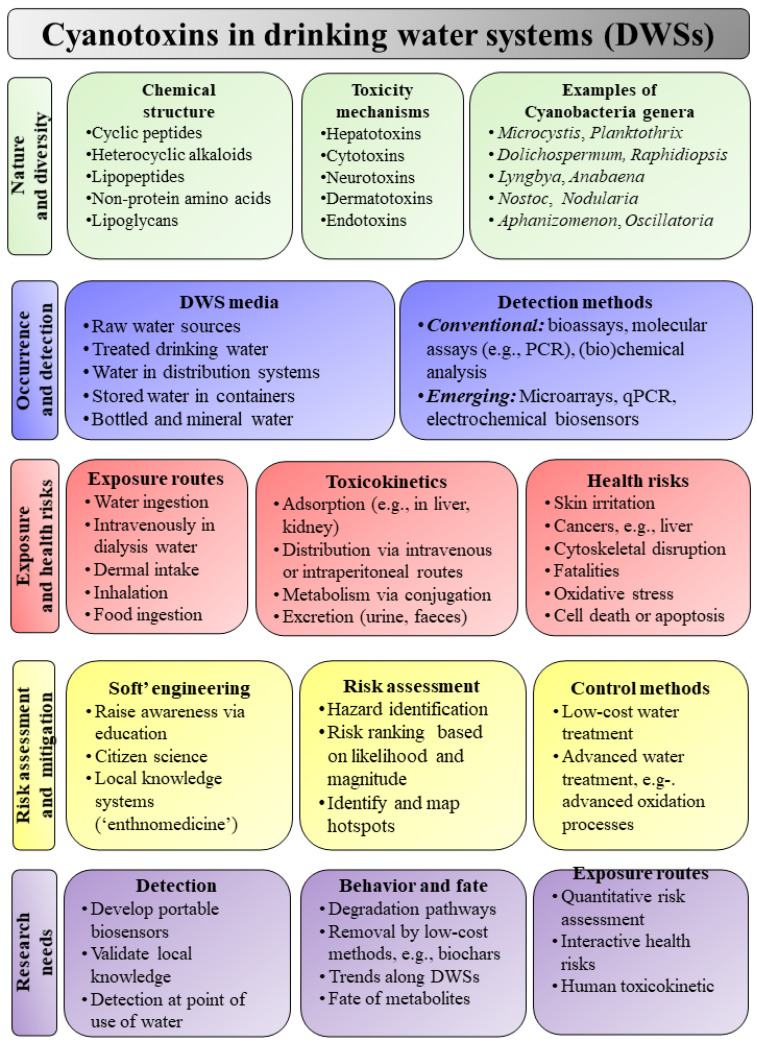
Conceptual summary of the nature and occurrence of cyanotoxins and their health risks and future research needs.

The present work aims to (1) examine the nature, sources, and diversity of cyanotoxins and their global occurrence along the source water–drinking water continuum, with a particular focus on low- and middle-income regions, (2) review evidence on human health risks, including exposure routes and high-risk populations, (3) explore toxicokinetics using the adsorption–distribution–metabolism–excretion (ADME) framework and mechanisms of human toxicity, (4) analyze the fate, behavior, and mitigation of cyanotoxins in DWSs, (5) propose a mitigation framework and outline ten key knowledge gaps, including the potential roles of ethnomedicine and ethnotoxicology, and (6) identify future research directions and priorities. The focus of this paper is summarized in Figure 1.

## 2. Methods

This study applied a generic semi-quantitative approach adapted from earlier reviews by our group [27]. The approach comprised (1) Boolean search techniques and (2) subsequent qualitative data analysis.

### Boolean Search and Retrieval Techniques

Boolean techniques were used to search and retrieve literature from English language scholarly databases (ScienceDirect^®^, Clarivate’s Web of Science^®^, Google Scholar^®^, Scopus^®^, and ResearchGate^®^). The focus on English language databases might have omitted some of these studies in other languages. However, given that English is the dominant language for scientific publication, it is believed that the literature and databases used accounted for the bulk of the articles. Particular attention was paid to recent articles covering a search period from 2000 to date, but earlier studies were included in cases where data were limited on particular aspects (e.g., Africa, human health risks).

The individual articles were then subjected to a preliminary qualitative screening and evaluation based on the study objectives. Nevertheless, there was limited evidence of drinking contamination by emerging contaminants before 2000. Therefore, subsequent searches were restricted to the period from 2000 to 2023.

Briefly, representative search strings for specific aspects were as follows:

***Occurrence of cyanotoxins in drinking water systems***

‘*cyanotoxin(s)* OR *algal toxin(s)* OR *microcystin(s) or harmful alga(e)’* AND *‘drinking water systems* OR *drinking water* OR *potable water* OR *water sources* OR *groundwater* OR *surface water* OR *stored drinking water’* AND/AND NOT ‘*Africa* OR *Europe* OR *North America* OR *Asia* OR *Australia* OR *Oceania* OR *south America* OR *Latin America OR Caribbea(n)’.*

***Cyanotoxin toxicokinetics***

*‘Cyanotoxins (and its variants)’* AND *‘Toxicokinetic(s)* OR *behavior* OR *ADME or adsorption–distribution–metabolism–excretion’* AND *‘human(s)* OR *human body* OR *mammal(s)* OR in vivo OR in vitro OR *preclinical* OR *rats OR mouse/mice* OR *pig(s)’.*

***Human exposure, toxicology, and health risks***

*‘Cyanotoxins (and its variant terms)’* AND *‘toxicity mechanisms (and variant terms* (e.g., *hepatotoxins, neurotoxins, dermatoxins, cytotoxins)’* OR *‘toxicology* OR *toxicological effects* OR *toxicity* OR *toxic effects* OR *health risks* OR *health hazards* OR *health effects OR health impacts* OR *health outcomes* OR *exposure pathway(s)* OR *exposure route(s)’* AND ‘human(s) OR rat(s) OR preclinical animals OR mammals OR mouse/mice OR pigs’.

***Behavior, fate, and remediation***

*‘Cyanotoxins (and its variants)’* AND *‘behavior* OR *fate* OR *degradation* OR *removal* OR *remediation* OR *treatment* OR *treatment process/methods’* AND *‘drinking OR drinking water* OR *potable water OR drinking water systems’.*

It is important to note that the presence of cyanotoxins in source waters (e.g., lakes, reservoirs, and rivers) was not assumed to directly imply contamination of formal drinking water systems (DWSs). Rather, our approach was to consider the broader source water–drinking water continuum, which includes both treated systems and regions where populations rely on untreated or minimally treated water sources. In many low- and middle-income regions, formal supply systems may be absent, and water is often collected directly from the environment for domestic use. We have therefore clarified in the manuscript that contamination in natural water bodies serves as a proxy for potential human exposure, with implications for both infrastructure-supported and informal water access scenarios.

The retrieved peer-reviewed articles were qualitatively screened for relevance to the study objectives, and relevant ones were retained for further analysis. Then, the relevant articles were reviewed in detail, while detailed tabulated results are in the Supplementary Materials (Table 1, Table 2, Table 3 , Table 4,  Tables S1 and S2). Note that there is a large body of evidence on cyanotoxins in aquatic systems. Therefore, the data in Table 1, Table 2, Table 3 , Table 4,  Tables S1 and S2 are not exhaustive. Instead, the data present an overview of the evidence drawn from several continents.

## 3. Cyanotoxins in DWSs

### 3.1. Diversity of Cyanotoxins

**Figure 2:**
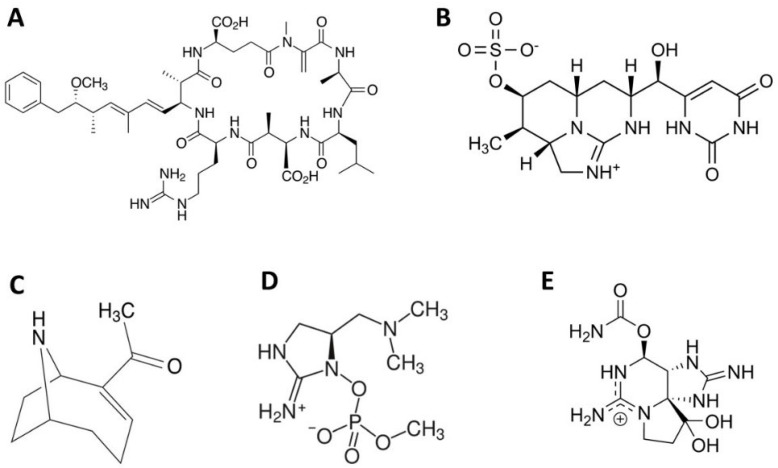
Chemical structures of (**A**) microcystin-LR, (**B**) cylindrospermopsin, (**C**) anatoxin-a, (**D**) anatoxin-a(S), and (**E**) saxitoxin (reproduced from [110] with permission from Elsevier).

Cyanotoxins can be categorized into five general groups: (1) neurotoxins (e.g., alkaloids anatoxin-a and saxitoxins, and non-proteinogenic amino acid β-N-methylamino-L-alanine), (2) dermatotoxins (e.g., lyngbyatoxin-A, aplysiatoxin), (3) cytotoxins (e.g., polyketide alkaloid cylindrospermopsin, CYN), (4) hepatotoxins (e.g., cyclic peptides, microcystins (MCs), and nodularins), and (5) endotoxins (lipopolysaccharides) [3,97,98]. Toxin production by cyanobacterial species is geographically diversified, and certain species can produce different compounds based on their climate zone. However, particular strains from the same region can also vary in this respect [99]. For example, *Raphidiopsis raciborskii* is a producer of CYN in Australia, but its strains in South America are producing saxitoxins instead, while in Europe, no known cyanotoxin-producing strains of this species have ever been identified [100]. The factors controlling this behavior may warrant further research. Additionally, different cyanobacterial species produce different cyanotoxins, leading to a wide range of structural variants even within a single toxin class. Several studies have revealed the existence of more than 90 different MC structures [101,102,103], while other studies have reported over 240 variants [104] and even higher (e.g., 250 variants in [105] and 279 in [106]). Anatoxins also have been reported to have different structures (Figure 2) [107,108,109]. There are over 25 chemical variants of saxitoxin [110], while CYN has a few analogs that differ in functional groups [111].

One should note that only a fraction of the toxic cyanobacterial metabolites are likely known. The CyanoMetDB, a comprehensive public database of secondary metabolites from Cyanobacteria, comprises over 2000 chemical compounds, most of which have not been studied concerning their toxic properties and mechanisms of action [112,113]. Various in vitro and in vivo studies demonstrate that cyanobacterial strains incapable of producing any known cyanotoxin still reveal significant toxicities in experimental models [43,114,115]. Therefore, cyanobacterial species shall not be unequivocally considered non-toxic based only on the lack of detection of known cyanotoxins or genes responsible for their synthesis. It also underlines the need to continue research devoted to cyanobacterial toxicities, including identifying toxic species/strains, the molecular basis of toxin synthesis and the environmental conditions influencing it, and understanding mechanisms of action and risks arising from exposure to environmentally relevant concentrations.

### 3.2. Global Occurrence of Cyanotoxins in Drinking Water Systems

Globally, cyanotoxins have been detected in DWSs in Africa, Asia, Europe, Oceania/Australia, North America, Latin/South America, and even Antarctica [116,117,118,119]. Detailed data on the occurrence of cyanotoxins in Africa and other regions outside of Africa are presented in Tables S1 and S2 in the Supplementary Materials, respectively. However, several countries in Africa and elsewhere still lack data. Specifically, while extensive research and regulatory frameworks are in place in many industrialized countries, the occurrence and contamination of DWSs by Cyanobacteria and cyanotoxins in regions other than Africa are underreported and poorly understood [120]. This subsection aims to provide an overview of the global occurrence of cyanotoxins in DWSs.

#### 3.2.1. Africa

Several studies have reported the presence of cyanotoxins and the associated cyanobacterial species in the water bodies that provide drinking water in the region (Tables S1 and S2 in the Supplementary Materials). The most frequent cyanotoxins detected are MCs. Of the 27 papers retrieved, all mentioned the presence of MCs in DWSs throughout Africa (Table S1). The presence of other cyanotoxins, such as anatoxins, CYN, and nodularin in DWSs has only been reported in a few studies conducted in only five countries (Botswana, Kenya, Tanzania, South Africa, and Zimbabwe) [121,122,123]. For instance, the neurotoxic anatoxin-a was found in Kenya’s Lakes Sonachi and Simbi at concentrations as high as 2.0 μg/g DW [121], and nodularin and CYN (0.004 to 0.01 µg/L) in Lake Victoria (Tanzania) [123].

According to studies [124,125,126], surface water in rivers, lakes, and reservoirs often contain higher concentrations of cyanotoxins, often beyond the drinking water recommendation guideline limit of 1 µg/L set by the World Health Organization (WHO), than other stages of the continuum. For example, six MC variants (MC-LA, MC-LR, MC-dmLR, MC-YR, MC-dmRR, and MC-RR) with concentrations ranging between 61.6 and 453.9 μg/L were detected in Ethiopia in the Legedadi Reservoir [12]. In a related study conducted in Zimbabwe, MCs with concentrations ranging from 18.0 to 22.5 µg/L were found in Lake Chivero [10].

However, many studies have shown a decrease in cyanotoxin levels from the source water to the point of use and consumption [11,125]. According to [127], most cyanotoxins are cell-bound and are easily eliminated by some water treatment techniques. For instance, in a Tanzanian study, no cyanotoxins were found in the piped water that had been treated. The water treatment techniques managed to eliminate the anatoxin-a, nodularin, and CYN found in the Lake Victoria source water [123]. Similarly, water treatment significantly decreased cyanobacterial cell counts and MC concentrations occurring at high levels in lakeshore water in Uganda [125]. However, there is little evidence that cyanotoxins can be eliminated or reduced to WHO-acceptable levels, especially in low-income settings in Africa. This is because this aspect has not been extensively investigated in low-income settings in Africa, where low-cost drinking water treatment processes such as chlorination, boiling, sand filtration, and solar disinfection are commonly used.

Several studies have detected cyanotoxins, in particular MCs, in various stages of the drinking water continuum at concentrations exceeding the WHO maximum guideline for human lifetime intake via drinking water [12,18,128]. According to [127], some water treatment methods, such as pre-chlorination and the use of algicides, cause the Cyanobacteria to lyse and release their toxins into the water. As a result, cyanotoxins may be present even in treated water. For instance, in a study carried out in Egypt, MCs (1–4 μg/L) were detected in the treated drinking water from the outflow tank [129]. Another study found MCs (4.3 μg/L) in South African treated tap water [130]. More importantly, it may be inferred that the conventional water treatment methods employed in some African countries are ineffective at removing or reducing the toxins to some acceptable levels, necessitating routine testing of treated water to give consumers access to safe drinking water.

Furthermore, it has also been observed that cyanotoxins can contaminate groundwater (Table S1). Some studies have revealed evidence that groundwater can become contaminated with MCs as a result of surface water and groundwater interaction [126,131,132]. This is a serious concern since many African communities rely on untreated groundwater for drinking, particularly from boreholes. Moreover, in many developing countries, residents often store treated drinking water in tanks or reservoirs, where cyanotoxins, particularly MCs, have been detected at concentrations exceeding the WHO guideline limit of 1 μg/L [62]. In South Africa, average values of 4.3 and 4.8 μg/L of MCs were found in communal tap water and treated water kept in tanks, respectively [130]. These results support regular toxicity monitoring of treated water stored in tanks, containers, and reservoirs.

#### 3.2.2. Asia

Detecting toxic cyanotoxins and Cyanobacteria in drinking water sources is an emerging phenomenon in the region (Table S2 in the Supplementary Materials). These problems have been noted mostly in China but also in countries such as India, Japan, and Turkey [133]. China has had major problems with cyanobacterial blooms and cyanotoxin contamination in its drinking water sources. Cyanobacterial blooms and cyanotoxins have been found in water sources across India, including Dal Lake in Srinagar [134] and Ambazari Lake and Phutala Lake in Nagpur [135], and thus they are no longer used as sources of drinking water. The screening of water reservoirs in Pakistan also revealed the presence of several MC congeners, with MC-LR and MC-RR being most prevalent, as well as the presence of CYN [136]. The toxicity of bloom-forming Cyanobacteria (*Raphidiopsis mediterranea*) discovered in Japan (Biwa Lake) has been experimentally proven [137,138]. In Turkey, several cyanotoxins (MC-LF, MC-LW, MC-LA, MC-RR, and MC-LR) were found in Kovada Lake, the concentration of which in the water reached 98.9 µg/L [139]. Cyanobacteria *Raphidiopsis raciborskii* and *Microcystis* spp. were recorded in the Myanmar Dam in 2020, reportedly producing cyanotoxins such as CYN and MCs [140]. Other studies have detected cyanotoxins in DWSs in Singapore, Vietnam, Thailand, Bangladesh, and Korea [141,142,143,144,145], Mongolia [146,147], as well as Iran, Saudi Arabia, and Qatar in the Middle East [148,149].

#### 3.2.3. Oceania/Australia

Cyanobacterial blooms and cyanotoxins in DWSs were a significant concern in Australia, especially from 1997 to 2003, when cyanotoxins were detected in many shallow lakes and reservoirs across the country. Concentrations of up to 18.9 µgL were recorded (Table S2). After this period, a serious incident was reported with the appearance of both saxitoxins and CYN in a large area (Murray River and its tributaries), which were used as sources of drinking water [150]. The serious threat to drinking water sources in Australia was the appearance of *Raphidiopsis raciborskii* in water bodies, releasing CYN into the water. In 1979, a major outbreak of gastroenteritis occurred in Palm Island, Australia, leading to the hospitalization of 10 adults and 140 children presenting symptoms of poisoning accompanied by elevated serum levels of liver enzymes, ketonuria, glycosuria, and hematuria. This case was linked to a massive *Raphidiopsis raciborskii* bloom in Solomon Dam, a drinking water reservoir, and the release of CYN toxin to the water column [151]. New Zealand has been affected by several incidents of cyanotoxins produced by *Raphidiopsis raciborskii* in the past [152,153,154,155], though the exact concentrations of this cyanotoxin are not known.

#### 3.2.4. Europe

Studies have detected cyanotoxins and cyanobacterial blooms in water sources across Europe. Cyanotoxins, such as anatoxins, saxitoxins, nodularins, MCs, and CYN, have been detected in lakes, reservoirs, and rivers used as drinking water sources (Table S2). In southern Europe (Serbia and Italy), the highest cyanotoxin (MC) concentrations of 650 µg/L were recorded in the Ćelije Reservoir and 75.5 µg/L in Alto Flumendosa Lake, while in Spain, the highest MC concentration was 64.8 µg/L in the Cogotas Reservoir [156,157,158]. Central Europe (mainly Poland and the Czech Republic) was most affected by cyanobacterial blooms and cyanotoxins (MCs). Algal blooms appeared for many years in several reservoirs (Siemianówka, Jeziorsko, and Sulejów dams), and cyanotoxin concentrations up to 173.8 µg/L were recorded in DWSs [159,160,161,162]. The problem of the intensity of inland water cyanobacterial blooms in Central Europe and its threat to DWSs was well described in Poland [163], where out of 238 water bodies analyzed, 204 were dominated by Cyanobacteria, and 74 water bodies were contaminated with cyanotoxins. However, only four of these water bodies were a source of drinking water [163,164]. There is little evidence of cyanotoxins occurring in DWSs in northern Europe. In Immeln Lake in Sweden, the highest concentration of cyanotoxins (i.e., MC) detected in drinking water was 815.0 µg/L [165,166]. There are also reports on the occurrence of cyanotoxins in Russian reservoirs [167,168,169] as well as toxic Cyanobacteria in Ukraine, though in the latter case, the information on particular toxins is limited to microcystins [167,170,171].

#### 3.2.5. South/Latin America

Besides Africa, South America appears to be the most affected continent in terms of the appearance of cyanotoxins in DWSs (Table S2). This is particularly evident in Brazil, where the greatest cyanotoxin (i.e., MC) concentrations in the water were as high as 303,053.9 µg/L in the Carpina Reservoir (Brazil) [172]. Recently, a report was published on the occurrence of MCs produced by *Limnothrix planctonica* in a source of drinking water in the Brazilian part of the Amazon River [173]. Although cyanotoxins appear infrequent on the continent, in Argentina, many drinking water sources have been reported to have been contaminated with MCs, nodularin, and anatoxin- [174,175]. In 1996, a fatal accident occurred in the Caruaru (Brazil) dialysis center. Lack of reverse osmosis in the filtration system resulted in the presence of MCs and CYN in the water supply, leading to the death of 76 exposed patients [38]. Cyanotoxins have also been detected in DWSs in several other countries in South America, including Colombia [176,177], Ecuador [178], Chile, Costa Rica, Dominican Republic, Mexico, El Salvador, Guatemala, Nicaragua, Panama, Uruguay, and Venezuela [179]. Some reviews also exist, focusing on the occurrence of cyanotoxins in aquatic systems in Latin America [180,181,182,183].

#### 3.2.6. North America and Antarctica

Cyanobacterial blooms and cyanotoxins in water bodies and DWSs are also a significant concern in North America (Table S2). Studies have reported potentially toxic cyanotoxins and Cyanobacteria in both treated and untreated water supplies used for household consumption [184,185]. Several reports on cyanotoxins in DWSs exist in the U.S., including those in state agency reports. One of the better-documented examples of cyanotoxins affecting drinking water is Lake Erie in the Great Lakes basin (USA), which provides drinking water for about eleven million inhabitants [186]. Since 2002, there has been a severe problem with the occurrence of MCs in water in this lake, and in 2014 an intense cyanobacterial bloom manifested [187]. In Mexico, however, anatoxin-a and CYN were detected in addition to MCs, with concentrations in the Los Berros water treatment plant reaching 129 and 7 µg/L, respectively [188]. In the United States, NOD was recorded alongside MCs, whereas in Canada, only MCs were recorded [187]. Cyanotoxins have also been detected in cyanobacterial mats in meltwater ponds in Antarctica [116,117,118,119]. This indicates the widespread occurrence of cyanotoxins and the ability of Cyanobacteria to survive under extreme temperature conditions.

### 3.3. Factors Controlling Cyanotoxins in DWSs

#### 3.3.1. Physical Factors Controlling Cyanotoxins in DWSs

##### Light and Temperature

Several studies have discovered that when light intensity is high, the Cyanobacteria produce more cyanotoxins, especially MCs, to protect themselves against reactive oxygen species [189,190,191]. It can be inferred that at low light intensity, the production of cyanotoxins is mainly supported by enhanced photosynthetic light reactions. Conversely, at high light intensity, algae are prompted to produce MCs as a response to photooxidative stress [192]. Moreover, various articles indicate that under increased temperature, the synthesis and release of cyanotoxins may be elevated [193,194,195]. However, there are also studies demonstrating that under decreased temperature, the MC quota per cell in M. aeruginosa can be increased [196]. In the temperate zone, the occurrence of Cyanobacteria associated with MC production was also noted during the winter season, indicating that winter blooms can be “cold and toxic” [197,198].

#### 3.3.2. Chemical Factors Controlling Cyanotoxins in DWSs

##### Nutrients

In addition to being essential for cyanobacterial metabolism, nitrogen and phosphorus also directly influence the production of cyanotoxins, and depending on the toxin, both starvation and overloading can cause increased levels of these metabolites [199,200]. More importantly, the synthesis of cyanotoxins is impacted differently by various types of nitrogen [201]. N and P enrichment of aquatic systems leads to eutrophication, a global environmental concern [202,203]. In turn, eutrophication increases the amount of nutrients accessible to Cyanobacteria, which might increase cyanotoxin production. In one study by [203], cyanotoxins were found in several lakes, with the highest overall concentration detected in the most eutrophic lakes in central Canadian ecozones. Their concentrations increased as cyanobacterial biomass increased, as did total phosphorus, dissolved inorganic carbon, and water temperature. However, it should be noted that oligotrophic water bodies have recently also become exposed to the risk of toxic blooms, for example, such as Lake Baikal, Lake Superior, or Alpine lakes [204,205,206]. Identifying the environmental factors and taxa that increase the occurrence of cyanotoxins is critical for the effective treatment of cyanotoxins in DWSs.

Several trace elements are also essential for algal growth, especially iron, copper, zinc, and sulfate. Studies have demonstrated that these elements are linked to the generation of cyanotoxins, though only at a specific concentration [199,201]. Pharmaceuticals and personal care products, endocrine-disrupting substances, pesticides, polycyclic aromatic hydrocarbons, and antibiotics are some of the more recent pollutants thought to control cyanotoxins’ emergence [207,208]. In addition to being a necessary micronutrient for Cyanobacteria to grow and reproduce, nitrogen is one of the main ingredients in cyanotoxins, such as MCs. For example, algal growth and the synthesis of cyanotoxins are compromised by extremely low or high nitrogen content. High phosphorus concentrations might indirectly alter the generation of cyanotoxins by affecting the biomass of Cyanobacteria [199]. However, nitrogen and phosphorus limitations can lead to an increase in the generation of cyanotoxins [201].

#### 3.3.3. Effects of Microbes on Cyanotoxins in DWSs

Cyanotoxins, such as MCs, are very stable compounds that defy physical breakdown at ambient pH and temperature [209,210]. However, certain microorganisms have been observed to break down MCs in water. These cyanotoxin-degrading bacteria are widely dispersed, and the degradation of cytotoxins by naturally existing bacteria is a viable option for toxin elimination [209,210]. As reported in several studies, the majority of cyanotoxin-biodegradation studies have been conducted on MCs, and several bacteria, such as probiotics and rumen bacteria, have been used to degrade MCs in DWSs at treatment plants [209,210,211]. These bacteria include *Arthrobacter* spp., *Brevibacterium* spp., *Burkholderia* spp., *Methylobacillus* spp., *Morganella morganii*, *Paucibacter toxinivorans*, *Poterioochromonas* spp., *Pseudomonas aeruginosa*, *Ralstonia solanacearum*, *Rhodococcus* spp., *Sphingomonas* spp., *Sphingosinicella microcystinivorans*, *Sphingopyxis* spp., and *Stenotrophomonas* spp. and have been reported to degrade MCs [209,210,211]. Despite claims of anatoxin-a biodegradation, only *Pseudomonas* has been demonstrated to digest anatoxin-a. In a study conducted by [211], it was discovered that *Sphingopyxis*, isolated from biological sand filters, played a crucial role in degrading MCs during water treatment processes. According to [212], 15 distinct fungal species exhibit significant lytic activity against Cyanobacteria, with 6 of these species capable of degrading MCs. Notably, selected fungal species, such as *Aurobasidium pullulans* and *Trichoderma citrinoviride*, have demonstrated the ability to specifically curtail the growth of Cyanobacteria while sparing beneficial algal species. Moreover, specific fungal strains like *Trichaptum abietinum* and *Trichoderma citrinoviride* have displayed a dual functionality, encompassing the lysis of Cyanobacteria and the breakdown of MCs produced from decaying cells [212]. Biological sand filters have been effectively utilized to purify drinking water by removing Cyanobacteria, cyanotoxins, and other microbial contaminants from the water supply.

#### 3.3.4. Effect of Seasonality and Hydrodynamics on Cyanotoxins in DWSs

Cyanotoxin variation during different seasons and variances in their quantities might be linked to a range of environmental conditions and cyanobacterial biomass [213]. As a result, monitoring during peak seasons is critical for preventing human health hazards. Research conducted in tropical Africa by [214], spanning the hot–dry, cool–dry, and hot–wet seasons, showed that Cyanobacteria relative abundances were highest during the cool–dry season. This phenomenon was likely attributed to a reduction in river inflows and an increase in reservoir mixing that tends to occur during the cool–dry season. Furthermore, the cyanobacterial community’s structure was significantly influenced by various factors, including macrophyte cover, dissolved oxygen concentrations, water quality, reactive phosphorus, water depth, and chemical oxygen demand [214]. The study by [214] emphasizes that potentially dangerous and toxic algae species may proliferate under these climate change projections.

A recent study [215] revealed that the Mexican dam had the highest cyanobacterial biomass compared to Chlorophyta and Bacillariophyceae throughout the year. The Cyanobacteria production was linked to the prevalence of cyanotoxin-producing species as well as environmental factors such as rainfall, total dissolved solids, water temperature, and certain trace metals. Similarly, [216] revealed that fluctuations in dissolved MCs and MCs bound to algal cells follow seasonal patterns, exhibiting a distribution with a single peak during specific seasons. Notably, the peak in dissolved MCs in water was observed to occur approximately one month after the peak of algal cell-bound MCs. However, according to [217], Cyanobacteria and cyanotoxins have a bimodal seasonal distribution, with seasonal fluctuation patterns differing across European climatic zones. This suggests that Cyanobacteria and cyanotoxins can follow the same trend in temperate and tropical regions. However, more studies using the same harmonized sampling techniques, sampling times, and methods for analysis are needed to understand which regions are prone to cyanotoxins.

According to [218], thermal stratification and a significant fluctuation in dissolved oxygen levels between the top and bottom were observed in October 2017 in the Itupararanga Reservoir (Brazil), and a decrease in cyanobacterial biomass was seen at the temperature-stratified sample locations throughout the same period. Visser et al. [219] state that temperature stratification favors floating Cyanobacteria due to reduced mixing and increased light availability. Although cyanobacterial biomass decreased in areas where thermal stratification occurred, [220] discovered that some species with aerotopes were abundant and that changes were also linked to light availability due to decreased water transparency, which might have a major effect on controlling dominance.

#### 3.3.5. Effect of Climate Change on Cyanotoxins in DWSs

Due to climate change, it is anticipated that Cyanobacteria will experience an increase in both their frequency and distribution, as this is likely to create favorable environments for their growth [221]. Furthermore, climate change’s impact might influence certain surface waters’ capacity to initiate phototransformation processes [221]. By fostering the perfect environment for Cyanobacteria to develop, climate change contributes to excessive cyanobacterial blooms and cyanotoxin production [222]. As reviewed by [223], human pollution and climate change cause Cyanobacteria to thrive, survive, and expand, potentially resulting in increased production of cyanotoxins. Warm waters are ideal for cyanobacterial growth; hence, as the planet’s temperatures rise, so do planetary water temperatures [222,223]. This is mainly because the topmost layer of the water stays considerably warmer than the rest of water column, which makes it more challenging for the water to mix, allowing Cyanobacteria to develop more efficiently [223]. Climate change-induced warming is increasing the thermal stratification of water columns. This confluence of variables (higher temperatures and stratification) supports cyanobacterial dominance, leading to cyanotoxin production [222,223]. This has the additional drawback of allowing this photosynthetic organism to readily take in sunlight and develop even more quickly, resulting in a thick layer on the water surface. Cyanobacteria can grow more readily when atmospheric carbon dioxide concentrations rise. The optimal environment for cyanobacterial growth, blooms, and cyanotoxin production is further created by increased water temperatures and carbon dioxide absorption [223]. Continental-wide analysis revealed that temperature is a major factor explaining the occurrence of cyanotoxins in Europe [224]. The frequency of cyanobacterial blooms during periods during which these events are usually not expected, e.g., in winter in the temperate zone, is also increasing [225].

Climate changes also impact precipitation rates and patterns. More storms may develop due to increased evaporation, although certain land regions will also be threatened by droughts [226]. Cyanobacterial blooms increase when more frequent nutrient contamination results from greater rainfall and storms [226]. Therefore, some actions that are accountable for the anthropogenic input of nutrients include fertilizing arable land, discharging sewage, using detergents extensively, and disposing of industrial effluents. Free et al. [227] discovered that the key characteristics determining cyanobacterial blooms in a shallow mesotrophic Mediterranean lake were seasonality, interannual fluctuation, lake level, water temperature, ocean oscillations, and concomitant rainfall. In addition, the same authors revealed that more extended periods of warmer temperatures can result in shorter-lasting blooms and subsequent production of cyanotoxins [227].

Vione and Rosario-Ortiz [221] conducted a study involving photochemical modeling in three distinct freshwater scenarios: (1) lakes undergoing summer stratification, (2) water exhibiting browning, and (3) water concentration due to evaporation. This investigation focused on two cyanotoxins, MC-LR and CYN, both characterized by distinct photoreaction kinetics and phototransformation pathways. Their results showed that the photodegradation of those cyanotoxins, which is significantly affected by reactivity with the triplet states of chromophoric dissolved organic matter, is quantitatively projected to be aided by anticipated changes in water-column conditions under changing environmental circumstances. This phenomenon, observed in the case of MC-LR, suggests that increased occurrence could be counteracted, to some extent, by accelerated photodegradation in specific environments [221]. However, the role of chromophoric dissolved organic matter in the degradation of other cyanotoxins remains poorly understood, indicating a knowledge gap in the intricate link between cyanotoxin photodegradation and changing climatic conditions [221].

According to a review conducted by [228], while climate change is predicted to enhance cyanobacterial blooms in some waterbodies, this is not the case in those with low concentrations of phosphorus or nitrogen. Such conditions meticulously restrict biomass, and even more so in shallower lakes and rivers than in stratified bodies of water. Limiting the release of nitrogen and phosphorus pollutants into water bodies is critical. In summary, instead of generalizations, management requires case-by-case evaluations focused on the implications of current and anticipated climatic changes on the specific waterbody, and these assessments should be carried out routinely and often as climate changes over time [228]. Moreover, it is crucial to understand and consider the fundamental factors and mechanisms driving the proliferation of Cyanobacteria and the production of cyanotoxins. This understanding is essential for formulating immediate and sustained strategies to mitigate global cyanotoxins’ increasing prevalence, severity, and health risks. As a result, it is prudent to develop an integrated nutrient management strategy that is consistent with different physical, biological, chemical, and integrated mitigation approaches, such as modifying water flow and flushing, dredging, applying chemicals, and integrating selective grazers.

### 3.4. Cyanotoxin Detection Methods

#### 3.4.1. Conventional Methods

Cyanotoxins have been detected using a range of conventional methods (Table 1) and are based on biological, biochemical, and physicochemical characteristics of cyanotoxins [34,229,230]. However, the criteria for selecting the method rely on sensitivity and specificity [230]. Initially, cyanotoxins were detected using methods involving extracts, cell cultures, and cyanobacterial cells injected in animal models [34]. However, such methods have since been discredited due to (1) high cost, (2) ethics surrounding animal models, (3) time-consuming procedures, and (4) no specificity and low sensitivity [133]. Therefore, more direct methods, including immunological assays, whole-organism bioassays, and biochemical assays, were subsequently designed [35]. However, whole-organism bioassays lacked sensitivity and specificity and could not be applied in rapid, regular water monitoring [34]. A good example of this is the mouse bioassay, which was qualitative and only revealed overall toxicity and did not reveal the identity or variant of the toxin. Other direct methods, like the Brine shrimp assay, protein phosphatase inhibition colorimetric, fluorometric, and radiometric detection assays, had shortcomings with sample matrix interferences and were less effective at detecting cyanotoxins in water samples [132].

Analytical methods that later became popular are based on the physicochemical aspects of the toxins: functional groups, ultraviolet chromophores, and reactivities. Such methods include gas chromatography (GC), liquid chromatography (LC), mass spectrometry (MS), nuclear magnetic resonance (NMR), capillary electrophoresis, Matrix-Assisted Laser Desorption Time-of-Flight Mass Spectrometry (MALDI-TOF-MS), and high-performance liquid chromatography (HPLC) [32]. HPLC-based methods, such as HPLC-MS and HPLC with the photodiode array, have been recommended for being highly sensitive and reliable, but like most analytical methods, they suffer from a lack of available standards and long derivation times; thus, they are time-consuming [32,35]. GC and LC methods are inexpensive and sensitive; however, equipment setup renders them less popular [35,133]. The majority of analytical methods may be suitable for MC detection in DWSs but are difficult to work with due to the co-occurrence of various cyanobacterial species within algal blooms as well as the production of many cyanotoxin varieties, which result in complex compositions [29]. To cater to these shortfalls, methods have been coupled to carry out a single analysis to quantify and identify MCs, such as LC/MS/MS.

Gas chromatographic methods are similarly significantly sensitive and precise but suffer from interference by compounds such as metal salts and organic and inorganic compounds. These interactions with sample components result in downstream cleanup processes such as solid-phase extractions becoming laborious and time-consuming [132]. In addition, chromatographic methods also demand highly skilled analysts, cumbersome, time-consuming procedures, rare standards, and, most importantly, expensive equipment and operations [34]. High-performance capillary electrophoresis efficiently separates and quantifies variants of cyanotoxins based on charge and molecular mass. Variations of capillary electrophoresis (such as those coupled with pressurized ultraviolet) have successfully detected MCs (low detection limit of approximately 0.92 to 2.3 µg/L), particularly in aquatic environments [31,34,231]. Attempts at lowering detection levels to trace concentrations by capillary electrophoresis have succeeded by incorporating immunoaffinity chromatography, field-amplified sample stacking, and dynamic pH junction. Despite the efforts, there is still a need to improve the detection limit with this method so that it can be well robust for routine water monitoring of cyanotoxins [232,233].

Recently, routine analysis in water has been prevalently performed using the cheap, easy-to-perform, qualitative, and semi-quantitative Enzyme-Linked Immunosorbent Assay (ELISA) for the detection of MCs/NOD, CYN, ATX, and STXs with limits as low as 4 ng/L [232]. The enzymatic immunoassay uses commercial kits to screen ground and surface water, detecting toxins produced by active and inactive Cyanobacteria genotypes. However, the method is delimited by a lack of uniformity of cross-reactivity, which makes it semi-quantitative. This is because measured concentrations of cyanotoxins depend on various variables that may not be recognized during analysis. Assay kits targeting an MC moiety, 3S-amino-9S-methoxy-2S,6,8S-trimethyl-10-phenyldeca-4E,6E-dienoic acid (known as ADDA), have been reported to generate false positive results in detection [234]. Other assays based on antibodies and protein phosphatases are available but are less popular because they fail to distinguish MC variants [28,231]. In order to overcome the limitations of conventional methods, very sensitive, selective, quick (responding in a couple of seconds), and fouling-resistant systems have since been developed.

#### 3.4.2. Advanced/Emerging Methods

Analytical methods that are robust and have now been designed to use micro-sensing devices, resulting in lower detection limits, improved target selectivity and specificity, and other benefits (Table 1). Biosensors are the latest technology that relies on the presence of (1) a bioreceptor in direct contact with a transducer and/or (2) a biological recognition element. These can be natural or artificial biological elements, such as antibodies, enzymes, nucleic acids, whole cells, and molecularly imprinted polymers [235]. Biosensors based on enzymes either rely on optical or electrochemical detection techniques. Those that employ antibodies are immunosensors, which can be detected via piezoelectric, NMR-based optical, and electrochemical sensors. Examples include immunoarray biosensors, luminescent immunosensors, Evanescent Wave fiber-optic immunosensors, surface plasmon resonance (SPR) immunosensors, and fluorescent immunosensors [36]. Those based on nucleic acids are electrochemical DNA and SPR-DNA biosensors.

Electrochemical biosensors achieve remarkably low detection limits due to the selective binding of a biological recognition element to the target analyte [32]. Two classes of electrochemical biosensors exist: (1) biocatalytic devices and (2) affinity sensors, which incorporate novel transducer and interface designs to yield remarkable results. These include metal and carbon-based composites that enhance electrode response [232]. Biocatalytic devices utilize enzyme target reactions to generate electroactive species, while affinity sensors, in contrast, oversee the interaction between the bioreceptor and the target to yield a quantifiable signal [232]. These sensors can employ labels for enhanced detection, categorized as ‘labelled’ or ‘label-free’ detection. Types of affinity sensors encompass aptasensors, DNA sensors, and immunosensors [29]. Biocatalytic devices utilize enzyme target reactions to generate electroactive species. In contrast, affinity sensors rely on the interaction between the bioreceptor and the target to yield a quantifiable signal [232]. These sensors can employ labels for enhanced detection, and thus can be categorized as ‘labelled’ or ‘label-free’ detection. Types of affinity sensors include aptasensors, DNA sensors, and immunosensors [29]. More recent technologies couple nanotechnology and biochemistry to increase signal transduction for detections as low as femtomolar concentrations.

Electrochemical biosensors are also categorized according to the mode of the electrical signal applied to the electrochemical cell. The categories include (1) potentiostatic (measures the current after controlled potential is applied to the electrochemical cell), (2) potentiometric (cell potential is measured under near-zero current), (3) impedance (potentially applied to the cell and the current response is measured to get complex resistance), and (4) galvanostatic (cell potential is measured following current application) [232]. In the past five years, impedance spectroscopy has gained popularity as it analyzes without the use of labels to create a detectable signal. The method is mostly preferred as it allows the study and interpretation of surface reactions and interface properties with simplicity during analysis due to its capacitive and resistive components. Examples of potentiostatic sensors are amperometry and voltammetry, and they have a dynamic range with low limits of detection. Voltammetric methods that have been used in detection are cyclic voltammetry, normal pulse voltammetry, differential pulse voltammetry, and square wave voltammetry [36].

One example of an electrochemical biosensor innovation with traceable efficiency is calf thymus DNA (ct-DNA), which was physically immobilized on a gold electrode following the discovery that MC-LR has a deleterious effect on ct-DNA. The method exhibited recoveries of around 107.6% while having detection levels as low as 1.4 ng/L in natural water samples [236]. Another novel method garnering popularity in automatic detection, particularly for onsite work, is the fiber-optical chemiluminescent biosensor system. The method utilizes a fiber optic bio-probe as both the biorecognition element, transducer, and Si-based photodiode detector, and its sensitivity achieves a low detection limit of 0.03 µg/L [237]. The method that uses a Surface-enhanced Raman Scattering (SERS) spectroscopic immunosensor that exhibits remarkable selectivity, sensitivity, and robustness is tailor-made for aquatic systems but operational with cultures of *Microcystis aeruginosa*. The SERS sensor attained a LOD of 0.014 µg/L [32,238].

The phosphorescent immunosensor utilizes antibodies and antigens as recognition units and Mn-ZnS RTP as sensing materials to bind specifically with MC-LR, rapidly detecting MC-LR at a low detection limit of 0.02 µg/L [32]. With this method, there is no significant obstruction from coexisting MC-LR pollutants in the water sample. In the detection of MCs in fresh crucian carp tissue and water, a novel method that uses Cu/Au/Pt trimetallic nanoparticles encapsulated in DNA hydrogel was prepared for colorimetric detection [32].

Aptasensors, an example of affinity biosensors that employ aptamers’ recognition element with high surface area and disposability, are now widespread. Nanoparticle technology has enabled the development of an aptasensor based on SERS. MC-LR aptamer and its corresponding complementary DNA fragments were conjugated to gold (signals) and magnetic (capture probes) nanoparticles. The method achieved a low detection limit of 0.002 ng/L. A low detection limit of 0.002 nM was achieved by employing a method that uses a dual signal amplification system with a ternary composite of AuNPs deposited on molybdenum disulfide (MoS_2_)-covered TiO_2_ nanobeads and horseradish peroxidase [133].

Another method utilizes a novel dual-mode aptasensor with MoS_2_-PtPd bimetallic nanoparticles and a zeolitic imidazolate framework (ZIF)-8-thionine (Thi)-Au (ZIF-8-Thi-Au) as signal material. A method based on non-radioactive energy transfer from an excited donor to an acceptor fluorophore, i.e., fluorescence resonance energy transfer (FRET), was utilized. The FRET-based method aptasensor positively correlated with the conventional ELISA method [133]. These methods are limited by temperature, pH, and ion concentration. A new sensor usable in real-world water systems was developed that uses a nanoprobe-based quantum-dot hapten sensing MC-LR at less than 0.03 µg/L. Interestingly, advanced fluorescent sensors have been developed that detect within 5 min and discriminate an array of cyanotoxins using smartphones. The technique integrates aptamers and single-stranded DNA dyes and has a low detection limit of less than 3 nM [232]. Aptasensor-based methods exhibit excellent recovery rates, indicating good stability against other components in water samples.

Quantitative real-time polymerase chain reaction (PCR) and DNA Microarray have improved sensitivity. They are based on the detection of the expression of toxin-encoding genes. Multiplex PCR is a recent alternative that amplifies and analyses many different genes in a single run [28]. This method is mostly suited for local authorities monitoring water bodies. Conventional PCR is only a qualitative (presence/absence) technique, while qPCR quantifies the reaction product in terms of gene copy numbers (with detection limits of 8.8 cells/mL of *Microcystis* spp.) [35]. The major limitation of qPCR is the reduction in amplification efficiency and the increasing length of PCR product. The presence of an amplified toxin-encoding gene does not necessarily indicate transcription, translation, or actual toxin expression. Therefore, it is essential to complement qPCR data with biochemical validation techniques. The microarray technique is a more recent high-throughput molecular method that screens for gene expression on a genomic scale. The technique is quick and efficient as it uses a chip loaded with DNA probes. Different adoptions of the technique enable gene expression studies using RNA and complementary DNA, and genetic variations using single-nucleotide polymorphisms or mutations. The major advantage of the microarray technique is the simultaneous analysis of the expression of many genes [34]. In one case, a microarray was used to simultaneously detect MC (*mycE*) and nodularin synthase genes (*ndaF*) expressed by hepatotoxin-producing Cyanobacteria. Similar studies were conducted using a microarray hybridized with a magnetic-capture hybridization technique that utilized bacterial magnetic particles. The technique is advantageous as it can be used as a monitoring strategy for cyanobacterial blooms in water systems; however, it is a costly method [35]. However, advanced analytical methods are often expensive, require high levels of skills to operate, and are often not readily available in low-income countries.

Potentially new toxins can be characterized while environmental monitoring can be carried out in DWSs by coupling existing emergent techniques with transcriptomic, proteomic, and metabolomic analyses. However, unlike indicator bacteria like *E. coli* and fecal coliforms, cyanotoxins are rarely included in routine drinking water quality monitoring, particularly in low-income nations. Furthermore, communities in distant and low-income areas frequently lack access to laboratory facilities for cyanotoxin testing. Therefore, there is a need to develop and validate low-cost analytical and detection tools to determine cyanotoxins in DWSs, including those based on (1) biosensors and (2) local knowledge systems (i.e., ‘ethno-toxicology, ethno-medicine’). In addition, further work is required to develop and apply maximum drinking water guidelines for total and individual cyanotoxins, especially in low-income countries where they are currently lacking.

## 4. Human Health Risks

### 4.1. Exposure Pathways

**Figure 3:**
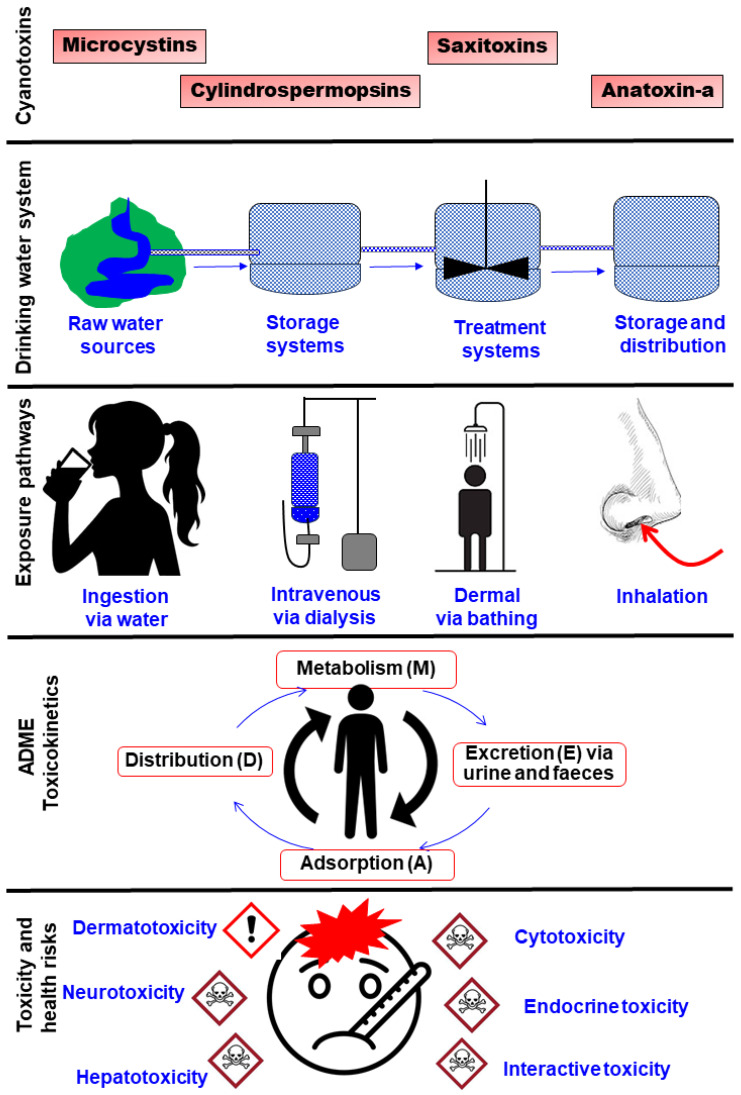
Overview of human exposure pathways, toxicokinetic processes, and health risks of cyanotoxins in drinking water systems.

Human exposure to cyanotoxins via DWSs is a cause for concern. Accordingly, several studies [98,239,240] have revealed human exposure to cyanotoxins through various pathways. Notably, ingestion (i.e., contaminated drinking water or food that has been prepared with it) and dermal exposure during routine activities such as showering, bathing, or swimming are all examples of exposure pathways (Figure 3) [241]. The global occurrence of cyanotoxins in various DWSs (Tables S1 and S2) makes human exposure to cyanotoxins difficult to avoid. For example, groundwater is typically extracted straight from the ground via a borehole to a standing tap and used for various activities without prior treatment [242]. The currently recognized exposure routes are skin contact (dermal route), inhalation, hemodialysis, and ingestion with water or food.

#### 4.1.1. Ingestion

Chronic and accidental ingestion of cyanotoxin-contaminated drinking water is the dominant route of human exposure to cyanotoxins. Direct ingestion is a frequent route of cyanotoxin intake, as evidenced by their occurrence even in treated drinking water. Several epidemiological studies conducted in various countries linked some diseases such as liver cancer (Serbia) [243], diarrhea, headache, vomiting, fever, muscular and abdominal pain (Sweden) [244], gastroenteritis (Serbia, Sweden, and Brazil) [38,244,245], and gastroenteritis-like ailments (Australia) [151,246] to consumption of contaminated water. Furthermore, exposure to cyanotoxins is also via ingestion of food prepared or cooked by contaminated water [247].

#### 4.1.2. Skin Contact (Dermal Route)

The skin has been identified as another route for cyanotoxin infection in humans, involving DWSs during bathing and recreational activities such as swimming. Recently, the ability of cyanotoxins, particularly those with small molecular sizes, to penetrate the skin has been demonstrated [248]. For instance, anatoxin-a, which has a log *p* value of 0.8 and a low molecular weight, may enter the body through passive diffusion through healthy skin. Some studies have also used human and animal models to assess the effects of cyanobacterial extracts on the skin [54,57,248]. Notably, 5–15% of people had significant skin reactions, as did the tested animals. In addition, irritant reactions on abdominal skin and ear tissue were shown, confirming the risk of dermal exposure. However, no quantitative dose–response relationship was reported. Studies in Australia indicated the occurrence of *Lyngbya majuscula* (a producer of lyngbyatoxin) bloom caused skin itching, redness, burning, blistering, and swelling in exposed marine recreational users [249].

#### 4.1.3. Intravenous (Hemodialysis)

The unique intravenous pathway via hemodialysis has placed humans at significant risk of exposure to cyanotoxins (Figure 3). The most fatal documented case of human cyanotoxin exposure occurred in Brazil, where out of 130 hemodialysis patients, 76 of 100 patients who developed acute liver failure lost their lives due to the use of water contaminated with MCs during their treatment [38]. The water employed for dialysis revealed a high concentration of MCs (19.5 µg/L), surpassing the World Health Organization’s safety threshold of 1.0 µg/L for drinking water supplies by 19.5 times [38,250,251]. A comparison of victims’ symptoms and pathology with data from animal studies led to the conclusion that intravenous exposure to MCs, specifically MC-YR, MC-LR, and MC-AR, was the major contributing factor to the mortality of dialysis patients [38]. However, the number of fatalities reported for the Brazilian incident seems to vary among studies, i.e., 52 in [251] versus 60 in [252] versus 76 in [38]. The source of this discrepancy is unclear, but could be attributed to the fact that some studies reported the total fatalities of all the people exposed to MCs via dialysis fluid, while others reported those who died due to specific human health conditions confirmed following an autopsy.

#### 4.1.4. Inhalation

According to [253], humans regularly breathe in aerosolized Cyanobacteria and cyanotoxins into their lungs. This is supported by recent studies that revealed that DWSs, particularly natural surface waters and artificial reservoirs, can produce bioaerosols that might contain various cyanobacterial water bloom components, which could potentially expose individuals through inhalation [254,255,256]. For example, cyanotoxin MCs (<35–415 fg/m^3^), endotoxins (0.13–0.64 EU/m^3^), cyanobacterial DNA (101–103 cells/m^3^) [256], and various Cyanobacteria-producing MC-LR species (maximum abundance of 1685 cells/m^3^) [255] were quantified in bioaerosols collected above surface waters. Similarly, Cyanobacteria and cyanotoxins have been detected in laboratory-aerosolized lake water [254,257]. As a result, the literature evidence such as the induction of respiratory irritation and inflammatory injuries [256], as well as the detection of Cyanobacteria in epithelial cells of the central airway and the upper respiratory tract [253,256], all point to bioaerosol exposure via inhalation. However, future studies are necessary to determine the extent of exposure in various DWSs along the continuum. More importantly, active water sports have also been identified as a poisoning pathway. Backer et al. [258] demonstrated that cyanotoxins can be transmitted from water to air by bursting bubbles on the water surface. The concentration of cyanotoxin in human breath samples was 2.9 ng/m^3^ compared to up to 5 ng/L detected in nasal swabs after participating in recreational activities on lakes containing 10–500 μg/L of MCs [42]. Koreivienė et al. [42] detected low MC concentrations in post-exposure nose swabs but not in blood samples, thereby confirming the inhalation route. In some cases, cyanotoxin exposure has also been associated with lung inflammation [259,260].

### 4.2. Potential High-Risk Settings and Groups

Identifying high-risk settings and groups for cyanotoxin is critical for decision-making, including devising, prioritizing, and implementing preventative and control measures. However, systematic studies investigating and characterizing settings and groups with a high risk of exposure to cyanotoxins and their health hazards at global, regional, and national scales are scarce. Potential high-risk settings and groups in low- and middle-income regions include (1) those relying on untreated drinking water from unsafe, polluted water sources, (2) countries where cyanotoxins are not routinely monitored in DWSs, (3) regions with high concentrations and diversity of cyanotoxins in drinking water at the point-of-consumption, and (4) healthcare settings where dialysis fluids with poor quality control and quality assurance are commonly used in hemodialysis patients. These high-risk settings and groups warrant attention in future research focusing on risk profiling and understanding the human exposure and health hazards of cyanotoxins.

### 4.3. Evidence of Human Health Risks

Human exposure to cyanotoxins is reportedly associated with a variety of illnesses, such as skin irritations, allergic reactions, gastrointestinal illnesses, liver failure, neurotoxic effects, and, in extreme cases, death (Figure 3, Table 2 and Table 3). Note that evidence on health risks of cyanotoxins is largely based on (1) clinical animal models such as mice, rats, and pigs, (2) indirect data on humans obtained from forensic and epidemiological studies, and (3) cases due to chronic exposure or teratogenic impacts on populations at risk [261]. The common health risk is gastroenteritis, resulting from consuming contaminated water [242]. Some of the earliest cases linking gastroenteritis to MC exposure were reported in 1931 in towns located along the Ohio River, USA [262], and from 1960 to 1965 in Harare, Zimbabwe [11,263]. Apart from directly consuming contaminated water, exposure to Cyanobacteria and their metabolites through recreational activities also causes gastroenteritis [132,264,265]. In addition, an increase in exposure time and the density of Cyanobacteria increases the incidence of gastroenteritis [264]. Contrary to the above findings, no significant hazards from exposure to cyanobacterial blooms in recreational waters were observed in some studies conducted in the United Kingdom and Australia [266]. The differences in results could be attributed to the species in the studied water bodies, which could have responded differently in the different environmental conditions, resulting in different toxicity levels.

Besides gastroenteritis, exposure to cyanotoxins may potentially be associated with liver cancers or chronic liver damage [47,245,267]. The International Agency for Research on Cancer classified MCs as Group 2B (possibly carcinogenic to humans), mostly due to their ability to disrupt cellular architecture and adverse effects on cell division and repair, as supported by the tumor promotion data [268]. Particularly, MC-LR exposure has been linked to the origin and progression of hepatocellular carcinoma and intrahepatic cholangiocarcinoma [269]. Other cyanobacterial metabolites were associated with allergic reactions, neurotoxic effects, and skin irritations [270]. However, the effects of long-term exposure to cyanotoxins are not fully understood. Scattered experimental and epidemiological studies correlated contact with cyanotoxin-contaminated water with various conditions affecting the liver [37,267]. Long-term consumption of cyanobacterial metabolites (e.g., in ng/L or µg/L levels), exclusively or in combination with other factors, causes chronic adverse effects such as liver cancers or chronic liver damage and diseases [37,267]. Laboratory-based experiments on children living in the Three Georges Reservoir Region in China assessed the relationship between chronic MC exposure and liver damage [38]. The results showed that chronic exposure to MCs can cause liver cell damage and ultimately significantly increase serum liver enzyme levels.

Neurotoxins are fast-acting compounds, and reports indicate that they interfere with the neuro-muscular system by inhibiting the transfer of excitatory signals, causing paralysis of the respiratory muscles, and death by respiratory failure within min to a few hours post-exposure [42,271]. For example, saxitoxins cause sensation loss by blocking sodium channels in nerve axons [271]. In addition, rat experiments have shown that MCs can cross brain barriers, accumulate in the brain, and cause changes in nerves and the brain [272].

Besides causing symptoms of varying severity, there are also reports of human fatalities related to cyanotoxin exposures [37,272,273]. The most tragic documented event incident in this regard occurred in 1996 in Caruaru, Brazil, when 76 patients died because of dialysate contamination by MCs [37]. The patients showed a host of conditions, including disruption of liver plates, mixed leukocyte infiltration, necrosis, liver cell deformity, cytoplasmic vacuolization, apoptosis, cholestasis, and multinucleated hepatocytes as well as intracellular edema, mitochondrial changes, rough and smooth endoplasmic reticulum injuries, lipid vacuoles, and residual bodies [262]. Although MCs were detected in blood serum and liver, further analyses implicated CYN in the poisoning, suggesting a possibility of multiple cyanotoxin exposures [274]. Consumption of cyanotoxin-contaminated portable water resulted in 2000 cases of gastroenteritis and 88 deaths in Bahia, Brazil, in 1988 [262,271]. Reports indicate that Dolichospermum and Microcystis genera Cyanobacteria had contaminated the water in the Itapirca Dam reservoir, but the studies did not identify the specific metabolites that caused acute gastroenteritis.

### 4.4. Behaviour and Fate of Cyanotoxins in the Human Body

#### 4.4.1. Cyanotoxin Toxicokinetics: The ADME Concept

The toxicokinetics of various cyanotoxins have attracted significant research attention [273,275,276]. Toxicokinetics describes the time-dependent behavior and fate of a toxicant (in this case, cyanotoxins) from the intake into the bloodstream, movement, or distribution to final excretion as a function of exposure dose or concentration (Figure 3). The ADME concept is widely used to assess toxicokinetics. Thus, four processes are central to the ADME toxicokinetic concept [276]: (1) absorption of the cyanotoxins from the exposure source into the human body or bloodstream, (2) the movement and distribution or partitioning of cyanotoxins from the blood into the tissues, including target organs and receptors, (3) the metabolism or (bio)chemical transformation of cyanotoxin into metabolites, and (4) the excretion rates or removal mechanisms of cyanotoxins from the human body. Some studies have investigated aspects of the toxicokinetics of cyanotoxins in humans and other organisms, including mammals such as rats, mice, and pigs [273,275]. However, for brevity, a comprehensive review of the toxicokinetics of all the individual cyanotoxins is beyond the scope of the present study. Here, we focus on the toxicokinetics of MCs as one example of the most toxic cyanotoxins often detected in drinking water systems. Here, the ADME concept is applied to discuss the evidence on four aspects of the toxicokinetics of cyanotoxins, with a focus on MCs as an example.

##### Absorption

Depending on the use of DWSs, cyanotoxins enter the human body mainly via the oral route [277]. However, at the point of use, DWSs serve multiple functions, including bathing, cooking, and medical applications (e.g., as a source of dialysis water). Thus, the exposure mechanisms could be complex and involve multiple sources and exposure pathways. In the literature, potential human exposure via inhalation and dermal intake has also been discussed [278]. Intravenous exposure via poorly treated dialysis water obtained from contaminated DWSs has been reported in healthcare facilities, including cases of fatalities (e.g., the Caruaru case in Brazil) [251]. Thus, absorption into the bloodstream may occur via the digestive and respiratory systems and intravenously. However, comprehensive information on the absorption of cyanotoxins in the human body is still lacking. The few available studies show low intestinal absorption of MCs. For example, one study using Caco-2 cells to understand the transport of MCs across the apical–basolateral pathway observed rapid uptake of 24 to 40% in the apical part, but only a small fraction (0.3–1.3%) reached the basolateral part [279]. This low efflux of MCs into the basolateral part is the key mechanism accounting for low intestinal permeability reported in other studies [280].

Other studies based on preclinical in vivo mammal models provide further insights into the absorption of cyanotoxins, particularly MCs. One study used a radio-labeled form of dihydro-MC-LR (MC-LR) administered orally to mice [281]. In the same study, the highest proportion of the MCs was detected in the liver (~0.68%), while the large and small intestines had nearly half the amount (i.e., ~0.25% each). About 38% of the radio-labeled dihydro-MC-LR was detected in the gastrointestinal tract contents, while the bulk of it (67%) was unaccounted for. These results point to the low oral bioavailability of MCs. Similar results were also observed in pigs exposed to MCs via gavage, where no MCs were observed in the serum following exposure to 2 µg/kg [275]. In the same study, about 1.1% of the total dose of the MC administered was detected in the liver, where it was covalently bonded to the protein phosphatases, while part of the unbound one was detected in the kidneys and large intestines. These absorption patterns control the distribution of cyanotoxins in the various organs and tissues. Besides studies using animal models, data on cyanotoxin adsorption in the human body are still limited. Moreover, the few available studies using preclinical animal models have focused on MCs, particularly MC-LR, possibly due to their frequent detection compared to other cyanotoxins. However, other MC variants (e.g., MC-LA, MC-RR, or MC-YR) often co-occur with MC-LR and have been detected in some studies [282,283,284,285,286]. In addition, although neurotoxic blooms are less common than hepatotoxic blooms, anatoxins and saxitoxins have been detected in Australia, North America, and Europe [287,288], and have been associated with deaths of wild and domestic animals [289,290]. Thus, besides hepatotoxins, neurotoxins could also be relevant to DWSs [289].

##### Distribution

Evidence on the distribution of cyanotoxins is predominantly based on studies conducted on preclinical animal species such as rats and mice [273,291]. These studies largely focused on MCs administered via intravenous or intraperitoneal routes. In earlier investigations conducted in the 1990s, the results showed that MCs bioaccumulate in the liver within about an hour after administration [281,292]. For example, about 71.5% of radio-labeled dihydro-MC-LR administered to mice via the intraperitoneal route accumulated in the liver within one hour [281]. A comparable accumulation of 67% within 1 h was also reported in mice following intravenous administration of MC-LR [292]. A few exceptions exist, where lower values were detected in the liver in other preclinical animal species. For example, one study showed that MC-LR distribution in Wistar rats, two hours following intravenous administration, decreased in the following order: muscle had the highest concentration (17.0%), then liver (6.1%), kidneys (3.7%), intestines (0.5%), lungs (0.3%), and finally gonads (0.1%) [291]. Another study based on intravenous administration of radio-labeled MCs showed that in albino rats, the distribution was highest in the liver (19.2%), followed by the gut contents (9.4%), and then the kidneys (5.3%) [293].

Studies have also investigated the half-life of MCs in preclinical animal species. One study showed that the percentage of MCs in the liver remained at 66% for six days after exposure, indicating a high retention time [292]. In other studies, half-lives of MCs of 0.8 min and 2.1 min for alpha particles and 6.9, 42, and 49 min for beta particles have been reported [293,294]. Further evidence based on LC-MS/MS analysis shows that the MC-LR concentrations in the liver remained unchanged two to seven days after exposure [295]. The long residence time indicates that MCs are bioaccumulated and are retained in the liver with minimal excretion. The bioaccumulation and long retention time in the liver may be due to (1) selective hepatic organic anion transporting polypeptide (OATP) transporters that promote transfer into the liver and (2) the absence of robust efflux pumps or transporters to excrete MCs from the liver [273,296,297]. Indeed, studies in both rodents and humans have demonstrated that hepatic OATP1B2 in rodents and hepatic OATP1B1 and OATP1B3 in humans facilitate the transport of MCs [296,297]. In mice, the Oatp1b2-null transporters were observed to be resistant to the hepatotoxicity of MCs, thus ensuring their continued functionality even in the presence of MCs [296,297]. On the contrary, one study showed that only one efflux transporter (MRP2) transported MC-LR out of a total of seven key efflux transporters investigated [298]. Therefore, the predominance of influx transporter systems for MCs relative to efflux transporters accounts for the high uptake and accumulation of MCs in the liver relative to their efflux.

A point to note is the difference in MC distribution among closely related rodent species (rats versus mice), but the reasons for this observation are unclear. However, differences among species have been reported in other studies (e.g., fish versus humans) and have been explained by several hypotheses related to differences in the capacity of MCs to bind to plasma proteins [299]. For example, one study observed that the LD_50_ of MC-LR in mammals was about 100 times lower than that of fish because the MC-plasma protein binding in the former was 16 to 28% compared to only 4% in the latter [299]. Further evidence shows that human albumin has the highest binding capacity for MC variants (MC-LR, MC-RR) compared to that of other species (e.g., bovine, porcine, and carp) [299]. Humans also have higher amounts of albumin than other species (e.g., fish). In addition, the facilitated uptake and dissociation of MCs bound to albumin, and possibly mediated by OATP transporters, may further explain the species differences [273]. The role of OATPs in the facilitated transport of MCs, even across the brain barrier, has been reported in some studies [300]. These differences among species raise questions about the transferability of toxicokinetic data based on non-human species often used in preclinical studies to humans. Further research is required to understand the role of albumin and OATPs in the transport, distribution, and partitioning of cyanotoxins in human organs. Such studies should move beyond MCs to include other cyanotoxins detected in DWSs.

##### Metabolism/(Bio)Chemical Transformation

The metabolism of cyanotoxins, particularly MCs, has been reported in the literature [273]. Metabolic pathways for MCs include the conjugation of glutathione (GSH) by glutathione-S-transferases (GSTs) to produce soluble and polar metabolites that are easily excreted [301]. In vivo studies show that the GSH conjugate undergoes further metabolism to a cysteinyl conjugate as the predominant metabolite [301,302,303]. Two human GSTs (GSTA1 and GSTT1) have excellent catalytic efficiency for MCs of 0.0012 and 0.0022 µM/min, respectively [304]. However, some in vivo studies suggest that the process is reversible, as evidenced by the detection of the parent form of MCs in tissues, urine, feces, and plasma following intraperitoneal injection of the GSH form of MC-RR [84].

MCs’ metabolic rate and overall metabolism appear low because the parent form of MCs is the dominant one detected in the liver following administration [295,305]. For example, a study conducted in rats showed the predominance of MC-LR in the liver in the first 24 h after administration, which dropped to about 50% of the total dose in three to seven days [295]. Another study based on LC-MS/MS also showed that MC-LR was still the dominant form detected in the liver two days following oral administration of MC-LR in rats [305]. A few exceptions exist—one study observed that about 83% of the radio-labeled MC-LR detected in the liver was covalently bonded to cytosolic components within 24 h. However, 83% likely included MC-LR metabolites and MC-LR conjugated to protein phosphatases [292]. In another study, higher MC-LR concentrations in the liver in rats relative to other organs were attributed to the inhibition of GSH synthesis, although this did not affect the concentrations of conjugate and GSH [306].

Evidence suggests that the toxicity of cysteinyl and GSH conjugates is less than that of parent MCs. In one study, the cysteinyl and GSH conjugates of MC-LR were 3 to 10 times less toxic concerning the inhibition of protein phosphatase than the parent MCs [307]. In another study, the LD_50_ values for cysteinyl and GSH conjugates of two MC variants (MC-YR and MC-LR) were 2.4 to 16.5 times higher than that of the parent MC following intravenous administration in mice [84]. Thus, the metabolic breakdown of MCs via the conjugation of GSH contributes to the detoxification of MCs. Further research is required to understand the human body’s metabolism and the metabolites’ toxicity based on typical concentrations detected in human tissue and organs.

##### Excretion

The excretion of MCs and parent compounds or metabolites occurs via feces and urine (Figure 3) [273]. The parent compound dominates the excreted MCs, followed by cysteinyl conjugate [84,308]. This indicates that the GSH conjugate of MCs is an unstable intermediate state that readily transforms to the cysteinyl conjugate or even back to the parent MC. One study showed that a total of 24%, comprising about 9% and 15% of radio-labeled MC-LR, was excreted via urine and feces, respectively, while about 83% bioaccumulated in the liver [292]. In the same study, 74% of the MC-LR excreted via urine occurred within 12 h, and approximately 63% and 30% of that was in the form of the parent compound and metabolites, respectively. The corresponding fecal excretion after 6 h and 12 h was 0.9% and 0.5%, respectively, but thereafter, the excretion dropped to only 1% per day for the next six days [292]. Comparable low urinary excretion of 1.9% of the total dose in 120 min was reported following intravenous administration of MCs in rats [293].

The fecal excretion of MCs following intraperitoneal or intravenous administration points to the biliary excretion of MCs [273]. This is further supported by data showing that, during 1 h liver perfusion, approximately 1.7% of radio-labeled MC-LR was excreted into the bile [309]. Still, the bulk of the MCs in the perfusate and bile excretion were in the form of the parent toxins, while about 85% of the toxins detected in the liver were in the polar form. Excretion of MCs in the bile has been reported in other studies using rats and mice [294,310]. The total amount of MCs excreted via feces and urine is relatively low, while the bulk of the MCs bioaccumulate in body organs, especially the liver. In light of this, options to minimize systemic exposure and human toxicity following exposure could be based on: (1) enhancing the metabolism or biochemical transformation to fewer toxic metabolites and/or (2) devising methods to enhance the excretion of both parent toxins and the metabolites.

#### 4.4.2. Toxicity Effects and Mechanisms

##### Individual Effects of Cyanotoxins

Several studies, including reviews, have investigated the toxicity mechanisms of cyanotoxins, particularly MCs (Table 3) [63,273,311]. Other studies have used omics techniques to investigate the toxicology of cyanotoxins [63]. Based on the toxicological or receptor target, cyanotoxins are classified into four main categories: (1) dermatoxins causing skin irritation on contact, (2) hepatotoxins acting on the liver, (3) neurotoxins causing injury or toxicity to the nervous system, and (4) cytotoxins causing both neurotoxic and hepatotoxic effects [312,313,314]. MC variants or congeners (MC-LR, MC-RR, and MC-YR) and nodularins are considered hepatotoxins [315]. MCs also damage the kidneys and reproductive system [233,238]. CYNs are cytotoxins that mainly target and damage the liver and nervous systems, as well as the kidneys, although they reveal a broad spectrum of adverse effects on various cells through the induction of oxidative stress [111,316,317]. Anatoxins (homoanatoxin-a, anatoxin-a(s), and anatoxin-a) are neurotoxins that target and bind the neuronal nicotinic acetylcholine receptors [318,319]. Aplysiatoxins and lyngbyatoxins are dermatoxins, while lipopolysaccharides are regarded as inflammatory substances [320]. Lyngbyatoxins are vesicatory and highly inflammatory substances, thus causing dermatitis and dermal lesions, and are also strong tumor promoters or carcinogens that activate the protein kinase C [321].

From a public health perspective, the broad categorization of cyanotoxins based on toxicological targets could be misleading. This is because most cyanotoxins have multiple toxicological receptors or targets rather than a single one. Specifically, several cyanotoxins have also been linked to other adverse health effects on histopathologic, cardiac, reproductive, gastrointestinal, and endocrine or hormonal systems (Table 3) [240,273,310,322,323]. Experimental studies and reviews on molecular toxicology have also reported several toxicity mechanisms for MCs alone, including (1) inhibition of protein phosphatases, (2) oxidative stress induced by reactive oxygen species, (3) cell death or apoptosis, and (4) cytoskeleton disruption [238,324,325]. MC variants are also well-known potent inhibitors for the protein phosphatase (PPP) enzyme system with the median lethal dose (LD50) of 0.1–1 nM for the PP1 and PP2A enzymes [49,326,327]. This inhibition of the PPP enzyme systems occurs via MCs covalently binding or bonding to the catalytic subunit of serine/threonine protein [326,327]. However, the extent of inhibition varies among MC variants as a function of the degree of methylation or demethylation status of the amino acids [328,329].

MC-induced oxidative stress is characterized by (1) excessive generation of reactive oxygen species, (2) depletion of GSH, (3) lipid peroxidation or malondialdehyde production, and (4) lactate dehydrogenase (LDH) leakage [330,331]. Thus, one study showed that administering an antioxidant to mice exposed to MC-LR mitigated hepatoxicity by reducing oxidative stress and downregulating the associated processes [332]. One study investigated the reproductive toxicity of MC-LR in mice and concluded that MC-LR significantly impaired spermatogenesis [333]. The possible mechanisms include (1) indirect or direct inhibition of the biosynthesis of gonadotropin-releasing hormones at the hypothalamic level and (2) a reduction in the luteinizing hormone concentration in serum. Collectively, the two processes suppress testosterone production in the testis. MCs also induce dose-dependent cell death, including apoptosis, necrosis, and/or autophagy [310,330,334]. For example, in HepG2 and Vero-E6 cells, MC-induced autophagy was detected at 25 µM and 50 µM, which are less than the critical threshold concentrations for inducing autophagy in HepG2 and Vero-EG cells, respectively [335]. In the same study, equal proportions of necrosis and apoptosis were detected at MC concentrations exceeding the corresponding critical threshold values. MC-induced cell death has been attributed to several mechanisms, including (1) oxidative stress, (2) inhibition of the protein phosphatases, and (3) disruption of the calcium homeostasis and cytoskeleton [264,336,337,338].

The cytoskeletal disruption caused by MCs involves the reorganization of microfilaments, microtubules, and intermediate filaments, which, in turn, lead to the deterioration in intercellular interactions and cell morphology [339,340]. Several mechanisms account for MC-induced cytoskeletal disruption: (1) oxidative stress or ROS generation and degradation of cell organelles and biomolecules, (2) the hyper-phosphorylation of cytoskeletal proteins, and (3) altered expression of cytoskeletal genes [322,341]. Several studies, including reviews on data in non-humans, particularly aquatic animals such as zebrafish, have also shown that MCs are endocrine-disrupting compounds (EDCs) [323,342]. A recent review of 61 studies showed that MCs meet eight out of the ten characteristics of EDCs in terms of interfering with six hormonal processes in the body with respect to (1) biosynthesis, (2) release, (3) circulation or distribution, (4) metabolism or biochemical degradation, (5) binding, and (6) action of natural hormones. The other two EDC characteristics associated with MCs were (1) epigenetic modification and (2) altering or disrupting the fate and behavior of the producing (source) and/or the responding (receptor) cells [323].

##### Interactive Effects of Cyanotoxins and Other Toxicants

In aquatic environments, including DWSs, cyanotoxins often occur as a mixture of diverse forms and chemical structures. These cyanotoxins also often co-occur with legacy and emerging contaminants, including organics, inorganics, and microplastics. Evidence shows that microplastics adsorb and act as vectors or carriers of MCs [343,344]. The interactions among cyanotoxins and between cyanotoxins and health stressors, including legacy and emerging contaminants, may give rise to complex synergistic and interactive toxicities or health effects. Limited studies have investigated the interactive human health risks of cyanotoxins and other human health stressors. One in vitro study investigated the genotoxicity and mutagenicity of a mixture of two cyanotoxins (MC-LR, CYN) and concluded that the toxicity of mixtures cannot be extrapolated from data based on individual cyanotoxins [51].

A study investigating the toxicity of a combination of chlorpyrifos (pesticide) and CYN on the differentiated SH-SY5Y human neuroblastoma cell showed dose-dependent individual and interactive effects of the two toxicants on viability and induction of necrosis and apoptosis [55]. Another study was conducted using mice to understand the interactions of MC-LR and cadmium exposures on the risk of chronic kidney disease as part of a case-control study in central China [345]. The results showed that exposure to MC-LR induced dose–response kidney injury in mice via the signaling pathway of phosphatidylinositol-3-kinase/protein kinase B/mammalian target of rapamycin (PI3K/AKT/mTOR). Comparison of the highest Cd dose quartile to the lowest value also showed dose–response effects of Cd. Moreover, positive additive or synergistic interactive effects were observed between Cd and MC-LR, indicating relative additional risk due to the combined effects of the two toxicants. The authors concluded that exposure to MC-LR was an independent risk factor for chronic kidney disease and had synergistic interactive effects with Cd, and both exhibited dose–response relationships. In one pre-print paper based on metabolomics and using the Asian clam as a bioassay species, a mixture of microplastics and MCs caused alterations in several metabolites in hepatopancreas compared to the control [323]. The authors concluded that these changes reflected perturbations in several molecular pathways, such as oxidative stress, amino and energy metabolism, and protein degradation/tissue damage. These early results highlight the need for specific toxicity evaluation of mixtures of cyanotoxins and even cyanotoxins and other contaminants.

The multiple toxicity mechanisms and receptor targets, coupled with the diversity of cyanotoxins in terms of origins and chemical structure, make the human health effects even more complex. The potential interactive health risks of cyanotoxins with other contaminants exacerbate this complexity. Due to their chemical diversity, data on toxicity and human health risks of cyanotoxins are limited to only a subset of the cyanotoxins dominated by MCs, which are the most common ones. Thus, information on the toxicity mechanisms of several cyanotoxins detected in DWSs is still limited. Further in vitro and in vivo research is required to better understand the interactive health risks of cyanotoxins with other human health stressors, risk factors, and underlying health conditions. Such information is critical for profiling and mitigation of public health risks.

#### 4.4.3. A Critique of the Evidence on Human Health Risks

Overall, available evidence on human health risks, including cyanotoxins’ toxicokinetics and toxicity mechanisms, has several drawbacks. For example, most of the data on toxicokinetics and toxicity mechanisms are based on extracted individual ‘pure’ cyanotoxins. Thus, such evidence fails to account for other co-occurring cyanotoxins and their interactions. Most toxicological studies tend to be short-term; hence, the results do not account for long-term and inter-generational health effects. Moreover, while human exposure to cyanotoxins is complex and involves multiple pathways, data on human health risks are often inferred from studies based on a single or a few exposure routes. For example, there is a lack of toxicological evidence on the ADME framework based on experiments simulating human exposure via dialysis fluids. Related to inter-generational health effects, the concept of Developmental Origins of Health and Disease (DOHaD) postulates that periconceptual, fetal, and early infant stages of life have an adverse impact on long-term health outcomes [346]. However, limited data exist validating the DOHaD concept for cyanotoxins. In real drinking water systems, cyanotoxins may exist as a complex mixture of various types and variants. However, few studies have partitioned the relative contribution of individual cyanotoxins in mixtures to human health outcomes. Moreover, given that some cyanotoxins have similar toxicity mechanisms while others have multiple health effects, this could create ‘equifinality’ and ‘multifinality’ problems. Multifinality implies that one contaminant may induce more than one human health condition or outcome, while equifinality implies that several emerging organic contaminants and even conventional pollutants may induce similar human health effects or outcomes [347]. The concepts of multifinality and equifinality complicate the interpretation of toxicological data of cyanotoxins but are rarely addressed in existing evidence. Note that these limitations are not limited to cyanotoxins but have been discussed in one of our earlier papers focusing on emerging contaminants in DWSs [347]. This calls for developing new conceptual tools and frameworks to address the complexity of understanding the human health risks of cyanotoxins in DWSs.

## 5. Risk Mitigation Strategy

A three-step framework based on hazard identification, risk assessment, and mitigation is proposed to mitigate cyanotoxins’ human exposure and health risks. A detailed discussion of related risk assessment and mitigation frameworks was presented in studies focusing on legacy and emerging contaminants [348,349,350]. Thus, for brevity, only an overview of the three key steps in the context of cyanotoxins is summarized here.

### 5.1. Hazard Identification

This entails identifying and characterizing the nature and hotspots of cyanotoxins in the drinking water value chain, including drinking water sources, water treatment systems, storage, and conveyance facilities, and at the point of use (e.g., households).

### 5.2. Risk Assessment

Risk assessment seeks to delineate high-risk settings, communities, and age groups (infants, elderly, and those with underlying health conditions) characterized by (1) prevalence of cyanotoxins (hazard) and (2) high likelihood (probability) of human exposure via DWSs. The risk assessment of cyanotoxins can be estimated using qualitative and quantitative risk assessment tools commonly used for other contaminants. Such risk assessment can be coupled to spatial GIS analysis, and the outputs can be visualized as spatial maps that can be used as decision-support tools.

### 5.3. Preventive and Control Methods

Several potential mitigation measures may be recommended depending on the socio-economic settings, including (1) adopting the precautionary principle in low-income settings lacking current drinking water treatment and data on the occurrence of cyanotoxins due to a lack of analytical laboratories. In such settings, the precautionary principle requires that one must assume the worst-case scenario and consider the drinking water as contaminated. Hence, mandatory drinking water treatment using the best available technology may be recommended. In cases where data are available, tools such as the Hazard Analysis and Critical Control Points (HACCPs), widely used in the food industry, and DWSs can be used for systematic risk assessment and mitigation/management [351,352]. Applying HACCPs could be part of a broader long-term strategy to develop drinking water safety plans at the regional or national levels. In light of the knowledge gaps highlighted, there is also a need for further research to better understand the health risks of cyanotoxins and their toxicity mechanisms. Such an understanding is critical for improved hazard identification and risk characterization. In cases where analytical laboratory facilities exist, cyanotoxins need to be included among routine parameters tested in drinking water, especially in regions with a known prevalence of harmful algal blooms. In such cases, drinking water should conform to current maximum guideline limits for cyanotoxins, and water violating such limits should be treated to achieve acceptable quality for drinking.

### 5.4. Removal of Cyanotoxins

Cyanotoxins have been removed from DWSs using a variety of techniques (Table 4). The cyanotoxin removal methods are classified into (1) conventional, (2) membrane-based methods/adsorption (carbon-based methods), (3) advanced oxidation technologies, (4) biodegradation, and (5) plant-based coagulants. Drinking water treatment with these methods can remove intracellular cyanotoxins (undamaged cyanobacteria cells) and extracellular dissolved toxins of several Cyanobacteria [69,83,94,129,353]. Although conventional methods have been shown to remove both extracellular and intracellular cyanotoxins, it was reported that methods such as flocculation/coagulation promote the release of MCs into the water [62,129]. Additionally, conventional MCs, which are typically applied post-flocculation/coagulation, such as chlorination, were reported to partially remove MCs and, therefore, result in concentrations of cyanotoxins (1.1 to 3.6 μg/L) above the permissible limits (1 μg/L) recommended by the WHO [69,129]. However, combining conventional methods and advanced methods has significantly reduced the cyanotoxins in drinking water. For instance, extracellular MCs are rarely removed from the water by coagulation, and it was reported that between 2.5 and 7.9% of MCs are removed by coagulation alone. When ozonation and coagulation were applied, between 6.8 and 11.7% of MCs were removed [67]. Moreover, chlorine and ultraviolet exposure led to lysis of 26.2% and 6.6% of *M. aeruginosa* cells, respectively. However, when ultraviolet/chlorine (with 0.2 mg/L of chlorine) was applied, the damage increased by 51.9% [69].

Various chemicals, such as oxidants, algaecides, chlorine, and permanganate, are commonly used in water treatment facilities to manage Cyanobacteria and cyanotoxins [354,355,356,357]. A crucial aspect of controlling cyanotoxins in DWSs involves reducing the availability of chemicals that Cyanobacteria require for growth. However, introducing an oxidant at the water intake presents challenges regarding cyanotoxin removal. One primary concern is avoiding the rupture of cyanobacterial cells. The consensus suggests that it is most effective to eliminate the maximum quantity of cyanotoxins while the cells are still intact and in the form of particles before the toxins are released [357]. Chlorine has demonstrated the ability to remove cyanobacterial cells, achieving a 90% removal rate within 10 min of oxidation [328,354]. However, limited data exist on the release and fate of the cyanotoxins following cell damage. Hence, the release and fate of cyanotoxins following cell damage warrant further research. On the other hand, permanganate does not react with CYN [354,356], and its reaction with anatoxin-a is rapid, although the rate is influenced by pH, with the rate doubling between pH 8 and 10 [358]. According to a study by [359], the effectiveness of permanganate oxidation in eliminating MCs depends on temperature, characterized by an activation energy of 16 ± 5 kJ/mol rather than pH (within the range of 6 to 10) or alkalinity.

Ozonolysis is generally quite a fast, effective process applied to eliminate cyanotoxins in DWSs. It uses the two oxidation mechanisms of ozone and hydroxyl radicals [360]. However, the extent of oxidation depends on ozone dose, temperature, and pH [360]. In addition, chlorine dioxide has been used to hydrolyze cyanotoxins in DWSs, but it was found to be ineffective at a concentration recommended for drinking water treatment. Most types of activated carbon have generally been effective in removing the most common and toxic cyanotoxins (MCs, anatoxin-a, and CYN) from DWSs. Even though the adsorption of cyanotoxins onto activated carbon varies by carbon type and source water chemistry, each application is unique; activated carbons must be tested to determine effectiveness [361,362]. Aluminum salts like aluminum sulfate are employed to control phosphorus levels in DWSs, limiting their availability as a nutrient for Cyanobacteria. Alum floc binds with sedimentary phosphorus, creating an aluminum–phosphorus compound that immobilizes phosphorus, reducing algae and enhancing water clarity as the floc settles. The effective aluminum dosage depends on water alkalinity, maintaining a pH between 5.5 and 9.0 [361,362].

Using algaecides to remove Cyanobacteria might not be optimal for reducing cyanotoxins, as they rupture cells, releasing higher cyanotoxin concentrations. This could decrease the efficiency of conventional filtration for cyanotoxin removal, requiring alternatives like activated carbon or oxidation [363,364,365,366,367]. Copper sulfate is a common algaecide due to its cost-effectiveness and ease of application, but its use raises concerns about sediment accumulation and harm to aquatic organisms. Chelated copper algaecides address this but pose similar environmental risks [363,364,365,366,367]. Peroxide-based algaecides may offer a relatively eco-friendly alternative to copper, while pollutants like metal ions, pesticides, and antibiotics also impact algal growth and cyanotoxin production [368,369]. However, peroxide-based algaecides may have potential adverse ecological health effects on non-target organisms. Moreover, the efficacy of peroxides depends on the physicochemical properties of the contaminated water [370]. For example, one study shows that *Microcystis* is highly sensitive to H_2_O_2_ at higher than lower light and under nutrient-enriched conditions [370]. Further research is needed to unravel the complex interactions among these factors affecting cyanobacterial growth and toxin production.

## 6. Future Perspectives

### 6.1. Harnessing Ethnomedicine and Ethnotoxicology of Cyanotoxins

There is a notable lack of studies documenting indigenous knowledge systems related to cyanotoxins, particularly in the fields of ‘ethnotoxicology’ and ‘ethnomedicine’ for mitigation strategies. Ethnomedicine refers to traditional medical knowledge and practices that interpret human health, disease, and healing within cultural contexts [371,372]. Ethnotoxicology, therefore, encompasses traditional knowledge on the health impacts of toxins, including cyanotoxins, on humans.

To date, research on ethnomedicinal and ethnotoxicological knowledge of cyanotoxins remains limited, even in low- and middle-income regions such as Africa, Southeast Asia, and Latin America, where traditional medicine and herbal remedies are widely used [373]. It is plausible that indigenous communities in these regions possess valuable knowledge on the health effects and potential mitigation of cyanotoxins. Therefore, documenting, validating, and integrating this knowledge into public health and water management strategies could enhance cyanotoxin risk mitigation, particularly in resource-limited settings.

### 6.2. Research Needs

Future research should focus on the following:

1. Understanding cyanotoxin occurrence, fate, and transport along the entire DWS chain—from the source to the point of use—including storage and conveyance infrastructure.
2. Investigating potential interactive human health effects of cyanotoxins with other pollutants, particularly emerging contaminants.
3. Further exploration of degradation pathways and their by-products, as well as the conditions of formation of these by-products and their toxicity compared to the parent compound.
4. Developing and evaluating novel, low-cost cyanotoxin removal methods, such as biochar and metallic iron, which have demonstrated efficiency in removing various water contaminants.
5. Exploring the potential methods to modify the cyanotoxins to regulate their toxic effects and make high-value products [374].
6. Process modeling of cyanotoxin occurrence, dissemination, fate, and behavior in DWSs to identify human exposure hotspots, especially in low-income regions.
7. Applying quantitative microbial risk assessment tools and disability-adjusted life years to profile cyanotoxin-related health risks in high-risk populations.
8. Developing and validating simple, cost-effective cyanotoxin indicators based on easily measurable water quality parameters, including those informed by indigenous knowledge, for implementation in low-income settings.

## 7. Conclusions

Cyanotoxins are emerging contaminants in DWSs, posing significant public health risks. Despite their widespread occurrence, research gaps remain in understanding their fate, human exposure, and mitigation strategies. This review provided a semi-quantitative analysis of cyanotoxin diversity, detection methods, health risks, and removal processes along the source water–drinking water continuum.

Evidence shows that cyanotoxins such as MCs, CYN, anatoxins, and saxitoxins are frequently detected in DWSs, with high-risk populations including communities relying on untreated water and those without routine monitoring systems. Advancements in detection technologies, including qPCR, LC-MS, and biosensors, have improved monitoring, but their accessibility remains a challenge in low-income settings. Conventional and advanced treatment methods show promise for cyanotoxin removal, yet cost-effective and scalable solutions are still needed, particularly in resource-limited regions.

Looking forward, key research priorities include (1) better characterization of cyanotoxin occurrence in storage, distribution systems, and at the point of use; (2) assessment of human health risks, particularly the impact of chronic exposure and interactions with other contaminants; (3) development of affordable and efficient removal strategies, leveraging both conventional and indigenous knowledge systems; and (4) exploration of ethnomedicine and ethnotoxicology as alternative approaches to understanding and mitigating cyanotoxin risks. Addressing these gaps will enhance global efforts to ensure safe drinking water and mitigate the health hazards posed by cyanotoxins. Future research should adopt a multidisciplinary approach, integrating environmental science, toxicology, public health, and traditional knowledge to develop holistic and sustainable solutions.

## Figures and Tables

**Table 1:** The summary of conventional and emerging methods employed to detect cyanotoxins.

Method	Key Capabilities	Merits	Demerits	Reference
Conventional PCR	Qualitative presence/absence test	Highly qualitative	Low sensitivity	[28]
Electrochemical biosensors	Analysis of low concentration of MCs	High recovery rates and low detection limits can be integrated into smartphones	Affected by temperature, pH, and ion concentration	[29]
ELISA	Detects 100 MC congeners	Applicable for field screening	Non-congener specific, low selectivity, cross-reactivity	[30]
GC/FID or MS	Uses gas chromatography	Inexpensive, sensitive	Affected by the co-occurrence of cyanobacterial species, requires equipment setup, and requires expertise	[30]
HPCE	Separates based on charge and molecular mass	Fast, easily applicable for the separation of cyanotoxin variants	Highly expensive	[31]
HPLC- (-MS, MS/MS, UV, PDA)	Can be coupled with other techniques for reliable, reproducible results	High congener resolution	Low selectivity, problematic quantitation, and high sample matrix interference	[32]
LC/ESI-MS/MS	Precisely and accurately identifies specific MC congeners	Low matrix interference	Limited standards	[33]
LC/TOF/MS	Precisely and accurately identifies specific MC congeners	Minimizes matrix interference	Limited standards for congeners	[33]
Microarrays	High-throughput gene expression studies, applicable in monitoring and tracking	Efficient, quick, and simultaneous analysis	High cost	[34]
PPIA	Specialized colourimetry for MC detection	Low instrument requirements	Cannot distinguish MC variants, low sensitivity	[23]
qPCR	Detects expression of toxin-encoding genes	Improved sensitivity	The presence of toxin-encoding genes does not translate to transcription, translation, and expression	[35]
Whole organism assay, e.g., mouse bioassay	May employ microbes, invertebrates, vertebrates, cell cultures, plants, and plant extracts	Can be calibrated against specific variants, highly qualitative	Low sensitivity, low specificity (no toxin identity), and not suitable for routine detection	[36]

ELISA—Enzyme-Linked Immunosorbent Assay; ESI—Electrospray Ionization; FID—Flame Ionization Detector; GC—gas chromatography; HPCE—high-performance capillary electrophoresis; LC—liquid chromatography; MC—microcystin; MS—mass spectrometry; MS/MS—tandem mass spectrometry; qPCR—quantitative polymerase chain reaction; PCR—polymerase chain reaction; PDA—photodiode array; UV—ultraviolet; and TOF—time-of-flight.

**Table 2:** Human health risks, including reported toxicity mechanisms following exposure via drinking water systems.

Cyanotoxin	Exposure route	Toxicity	Mode of Action	Reference
Microcystin (MC)	Reservoir water	Toxin covalently binds and inhibits catalytic subunits on protein PP1 and PP2A, causing hyper-phosphorylation of cellular microtubules, hence loss of cellular structure.	MC-LR daily intake of 2.03 µg/L was associated with liver damage in children. Causes cellular apoptosis.	[37]
MC	Water used for dialysis treatments	MCs disrupted the liver plate pattern. MCs are inhibitors of eukaryotic protein serine/threonine phosphatases 1 and 2A.	Liver cell damage	[38]
MC	Contact and swallowing MC-contaminated reservoir water	Pulmonary intravascular formation of protein deposits and removal of platelets from circulation.	Liver damage, pneumonia, and pulmonary thrombosis. Death.	[39]
Microcystin-LR (MC-LR)	Injection	Intrahepatic hemorrhaging. MC-LR covalently binds to protein phosphatase 1/2A.	MC-LR dose-dependent hepatocellular hypertrophy, degradation and necrosis. Hepatic inflammation. Fibrosis in the livers of rats.	[40]
MC-LR plus thioacetamide (TAA) (MC-LR/TAA)	Drinking contaminated water	Inhibits serine/threonine phosphatase activity, enhances proliferative activity, or activates HSCs to transform to a myofibroblast phenotype.	Liver inflammation.	[41]
Saxitoxin	Contact with water during recreational activities	Blocking sodium channels in nerve axons. Inhibits the transfer of excitatory signals.	Causes paralysis of the respiratory muscles and death by respiratory failure.	[42]
Cylindrospermopsin (CYN)	Contact and drinking CYN-contaminated water	Causes oxidative stress in human neutrophils, eventually leading to lipid peroxidation and decreased cell survival.	Apoptosis in human T-lymphocytes.	[43]
CYN	Intraperitoneal administration	Causes oxidative stress in human neutrophils, eventually leading to lipid peroxidation and decreased cell survival.	Human hepatoblastoma and human colon adenocarcinoma cells.	[44]
MC	Drinking contaminated lake water and food	MC-LR leads to hepatic steatosis with molecular alterations in circadian rhythm regulation, lipid metabolic processes, and the cell cycle pathway.	Liver damage and lipid metabolism dysfunction. Long-term exposure leads to non-alcoholic fatty liver disease (NAFLD)	[45]
MC	Injection	Damages diabetes genes and other proteins, e.g., Ppp3ca, Ide, Marcks, Pgk1, and Ndufs4.	Increased risk of diabetes in humans. Reduction in blood insulin levels.	[46]
MC-LR	Human hepatocellular carcinoma cell line (HepG2) was exposed to solutions of MC-LR	Under normoxic conditions, MC-LR stimulates the proliferation of the HepG2 cell line, promoting liver tumor and liver cancer cell growth. Under hypoxic conditions, MC-LR induced apoptosis in the HepG2 cell line by inducing prolonged oxidative stress.	Liver cancer	[47]
MC-LR	Oral intake of MC-LR-contaminated water	MC-LR causes alterations in TBARS, superoxide dismutase (SOD) activity, and glutathione content in the liver and intestine of mice.	Exposure to 50 mg MC-LR/kg every 48 h generates significant damage to the liver and intestine.	[48]

**Table 3:** General toxicity, including target organs and receptors of cyanotoxins.

Cyanotoxin	Receptor Organ, Tissue or Process	Toxicity	Reference
Hepatotoxicity			
Microcystin-LR (MC-LR) and dehydrobutyrine (Dhb)-containing MC variant [Asp3, ADMAdda5, Dhb7] MC-HtyR isolated from *Nostoc* spp.	In vivo phosphoprotein phosphatase (PPP) family (PP1, PP2A, PPP4, and PPP5)	Dhb-containing MCs and MC-LR inhibited PP1, PP2A, PPP4, and PPP5. MC-LR formed a covalent bond with a cysteine residue in the PPP, while Dhb-MC-HtyR did not form any covalent interaction with PP2A. Nostocyclin, a cyclic peptide also isolated from *Nostoc* spp., was non-toxic with more than 500-fold less inhibitory effects on PP1, PP2A, PPP4, and PPP5. Given that LD50 for MC-LR was lower than that of Dhb-containing MCs and nostocyclin, the inhibition of protein phosphatases seems to be the most harmful toxicity mechanism of MCs in vivo.	[49]
Microcystinss (MCLR, LA, LF, LW, LY) with changed Z^4^ residues	Protein phosphatase 2A	MC (MCLR, LA, LF, LW, and LY) inhibition effects on PP2A were explored by a colorimetric protein phosphatase inhibition assay. The inhibition sequence of MCs on PP2A was detected as follows: MC-LR > MC-LW > MC-LA > MC-LF > MC-LY.	[50]
MC-LR (MC-LR) and Cylindrospermopsin (CYN)	*Salmonella typhimurium*, L5178Y Tk^+/−^ cells, and Caco-2 cells	An in vitro battery was used to assess the mutagenicity and genotoxic potential of a mixture of MC-LR and CYN. The in vitro mutagenicity and genotoxicity showed by CYN/MC-LR mixtures do not differ substantially from that observed for CYN tested individually. The results indicate that cyanobacterial mixtures require a specific (geno)toxicity evaluation as their effects cannot be extrapolated from those of the individual cyanotoxins.	[51]
MC-LR	Microfilament depolarization and expression of microRNA-451a (miR-451a) in HL7702 liver cells	Data demonstrated that MC-LR increased microfilament depolarization, elevated phosphorylation levels of mitogen-activated protein kinase (MAPKERK1/2) and vasodilator-stimulated phosphoprotein (VASP), but lowered miR-451a RNA expression levels. Data demonstrated that transfection with miR-451a may not be effective in the presence of MC-LR, as evidenced by the inability of excess microRNA to prevent toxin-induced inhibition of threonine protein phosphatases 1 (PP1) and 2A (PP2A) and microfilament reorganization in HL7702 cells.	[52]
Neurotoxicity			
CYN	The skin of albino Californian rabbits	Edema and erythema were observed after 24 h following intradermal exposure to 0.2 mL of lyophilized *Umezakia ovalisporum* (formerly *Aphanizomenon ovalisporum)* extract, and skin irritation was rated moderate.	[53]
CYN	Abdominal skin of Balb/c mice exposed to 100 µg of CYN mL^−1^ via topical application	Yellow/brown crusts, dried skin, blood, or serous fluid oozing from exposed skin and desquamation were observed. Skin lesions were observed starting from the second induction day onwards. Cell infiltration predominantly of the mononuclear cells, edema, inflammation, and thickening were noted on the ears after 24 h and 48 h of exposure. At an exposure dose of 73 µg CYN/mL ear swelling was observed.	[54]
Cytotoxicity			
Interactive cytotoxic effects of CYN and a pesticide (chlorpyrifos)	Differentiated SH-SY5Y human neuronal (neuroblastoma) cells	A concentration-dependent decrease in viability was observed for the contaminants. Interactive toxic effects induced signs of necrosis and apoptosis, but acetylcholinesterase activity appeared unaffected. The results demonstrate the need to understand the interactive toxic effects of cyanotoxins and other health stressors.	[55]
Variants of cyanobacterial extracts (crude MC-containing, purified MC-containing, and non-MC-containing extracts)	Cultured human lymphocytes	The effects of MC-producing Cyanobacteria on human lymphocyte culture and their potential for adverse human health effects were investigated. The study indicated the highest cytotoxicity and genotoxicity in the purified extract containing MCs (MC-LR, MC-RR, and MC-YR). No significant effect or clear relation between MC concentration in crude extracts and human lymphocyte mortality and DNA damage was observed. This research supports the necessity of the application of additional tests for the determination of total threat from natural cyanobacterial blooms, apart from the determination of concentration and toxicity of the main group of cyanotoxins.	[56]
Dermatotoxicity			
Extracts of *M. aeruginosa* (non-toxic strain), *A. circinalis*, *N. spumigena M. aeruginosa* (toxic strain), *A. incerta,* and *C. raciborskii*	Skin of human volunteers	The results showed that between 10% and 40% of both atopic and non-atopic individuals showed adverse reactions to at least one of the active patches of each of the six cyanobacterial species and concentrations investigated. A small number of participants showed evidence of induced erythema via skin contact, but no dose-dependent or threshold behavior was evident.	[57]
Miscellaneous toxicity effects			
MC-leucine arginine (MC-LR)	DNA	The direct effect of MC-LR on DNA was explored by using spectral analysis and molecular biotechnology. Spectra analysis and molecular biotechnology showed that there is no direct interaction between DNA and MC-LR This study discovered that the effects of MC-LR on DNA originate mainly from the secondary effects of MC-LR rather than from the direct interaction between DNA and MC-LR.	[58]
MC-LR	Interaction of MC-LR with proteins and DNA	Experiments were performed to explore the interaction of MC-LR and proteins. The results indicated that there is binding of MCs to a wide range of proteins in vitro. Under the conditions tested, MC-LR does not exhibit affinity for DNA.	[59]
Combination of serum MCs, plasma As, and Cd	Human kidneys	The association between MCs, As, and chronic kidney disease (CKD) was investigated using conditional logistic regression. The results showed that MCs and As were significantly associated with CKD risk. Both MCs and As are independent risk factors for CKD, exerting a synergistic effect between them. Combined exposure to MCs, As, and Cd concentration can increase the risk of CKD.	[60]

**Table 4:** Summary of the methods employed to remove cyanotoxins from drinking water systems in different world regions and their effects.

Cyanotoxin	Receptor Organ, Tissue, or Process	Effect	Reference
Conventional methods			
North America	Coagulation and sand filtration	Reduced intracellular cyanotoxins by 59–97%, with most MC removal occuring during coagulation, filtration, and settling.	[61]
Egypt	Sedimentation/ sand filtration and coagulation (flocculation)	Sedimentation, coagulation, and filtration removed 91–98.9% of MCs, but flocculation may release MCs. Chlorination partially degraded toxins, sometimes exceeding WHO limits.	[62]
Egypt	Coagulation, sedimentation, and flocculation	Removed various toxin-producing Cyanobacteria, but *O. limnetica* cells were only partially removed.	[62]
China	Coagulation (flocculation) with flocculants and coagulants, including ferric chloride and aluminum salts	Removal of cyanotoxins by 90% from drinking water.	[63]
Canada	Clarification, coagulation, and filtration	Cyanotoxin-producing *Aphanizomenon* cells were poorly removed by coagulation. *Pseudanabaena* and *Anabaena* were efficiently removed from the water by filtration and clarification.	[64]
Canada	Coagulation	90% of intracellular MCs were removed. Removal of sludge after coagulation was recommended to avoid the re-release of MCs to water.	[65]
Egypt	Rapid sand filtration and disinfection methods	Rapid sand filtration of drinking water partially removed *Microcystis* and *Pseudanabaena limnetica* (formerly *Oscillatoria limnetica)* (ranging from 25 to 34%). *O. limnetica* cells were reported to resist disinfection, which is applied in the final stages of water treatment, and disinfection only reduced *O. limnetica* by between 25 and 66.7%.	[66]
Portugal	Ozonation and coagulation	2.5–7.9% and 6.8–11.7% of microcystins (MCs) were removed by coagulation before and after ozonation, respectively.	[67]
China	Ferrate oxidation	Ferrate oxidation removed up to 98% of cyanotoxins, with efficiency influenced by contact time, dosage, and pH. A 30 min contact period at 40 mg/L ferrate and pH 6–10 yielded optimal results.	[68]
China	Chlorination and UV application	Chlorine (0.2–2.0 mg/L) and ultraviolet (UV) treatment damaged up to 96.6% of M. aeruginosa cells and degraded microcystin-LR (MC-LR_ by 69–83%. Combined UV/chlorine treatment was most effective with removal percentages of MC-LR between 68.9 and 82.7% in 15 min.	[69]
Belgium	Dissolved air flotation	Air flotation removed 40–80% of MCs, 90–100% of Anabaena, and 30% of Planktothrix cells.	[70]
Canada	Sand and graphitized sand filtration	Graphitized sand filtration reduced MC-LR to <0.61 μg/L (within WHO limits), outperforming standard sand filtration at a lower cost (CAD 160 vs. 6000).	[71]
France	Rapid and slow sand filtration	Bacteria-assisted filtration removed up to 99% of algal cells and toxins, offering a low-cost potable water solution.	[72]
South Australia	Biological sand filtration	MC-LR and microcystin-LA MC-LA were completely removed from drinking water.	[73]
Membrane-based methods/Adsorption (carbon-based methods)			
Germany	Ultrafiltration	Membranes removed 98% of intracellular MCs and >96% anatoxin-a, eliminating both toxins and toxin-producing microbes.	[74]
Canada	Granular activated carbon (GAC) and powdered activated carbon (PAC) adsorbents	GAC reduced MC-LR from up to 47 to 1 μg/L; PAC (100 mg/L) removed 86%, lowering MC-LR from 22 to 3 μg/L.	[75]
South Africa	PAC (tyre-based	Tyre-based PAC achieved 100% MC-LR removal at pH 4, 10,000 mg/L carbon, in 34 min.	[76]
Switzerland	Gravity-driven membrane (GDM)	GDM systems removed M. aeruginosa and lowered cyanotoxins to <1 μg/L within 15 days.	[77]
Australia	Microfiltration and ultrafiltration	Membranes removed >98% of intracellular M. aeruginosa cyanotoxins.	[78]
China	Nanomaterial algacidal sheet	Algaecidal sheets removed 56–89% of *M. aeruginosa* and *Anabaena* within one day.	[79]
Australia	GAC	GAC removed up to 95% of anatoxin and extracellular MCs.	[80]
Brazil	GAC coupled with Fenton oxidation/flocculation/coagulation/sedimentation	GAC columns adsorbed 4.15 μg/g MCs; combined Fenton oxidation and GAC effectively purified water to safe limits.	[81]
Germany	Ultrafiltration	Membranes removed 98% of MCs and >96% anatoxin-a, eliminating toxins and producers.	[74]
Canada	Granular activated carbon (GAC) and powdered activated carbon (PAC) adsorbents	GAC decreased MC-LR to 1 μg/L; PAC (100 mg/L) reduced MC-LR from 22 to 3 μg/L.	[75]
South Africa	PAC (tyre-based)	Tyre-based PAC removed 100% MC-LR at optimal conditions (pH 4, 10,000 mg/L, 34 min).	[76]
Advanced oxidation technologies for the degradation of cyanotoxins			
Canada	Potassium permanganate oxidation	Up to 95% of MCs were removed from drinking water.	[65]
China	KMnO_4_ pre-oxidation	MnO_4_ combined with sand filtration or Mn-oxidizing bacteria (e.g., Pseudomonas) enhanced the removal of MC-LR and BP-4 via transformation of Mn^2+^ to Mn oxides.	[82]
China	•OH oxidation	•OH (1 mg/L) pre-treatment, alone or with 0.5 mg/L NaClO, inactivated blooms and maintained disinfection byproducts (DBPs) within Chinese drinking water safety limits.	[83]
China	•OH oxidation	In a 480 m^3^/day plant, •OH (0.88 mg/L) rapidly inactivated algae; combined with ClO_2_ and sand filtration, it met all Chinese water quality standards.	[84]
German	Ozonation	Ozone (1.5 mg/L) degraded *M. aeruginosa* toxins; as little as 0.2 mg/L oxidized MC-LR below detection within seconds to minutes, reducing toxicity.	[85]
Australia	Ozonation	Ozone (0.2 mg/L) degraded MC-LR below high-performance liquid chromatography (HPLC) detection limits within a short period of time (sec to min).	[86]
United Kingdom	TiO_2_ and UV	TiO_2_ and UV removed MCs in under 45 min.	[87]
China	Ferrate (VI) and UV	Ferrate (VI) (0.08–0.17 mmol/L) with UV degraded up to 100% of MC-LR.	[88]
Biodegradation			
Egypt	Biodegradation by *Bacillus* sp.	Up to 300 μg/L of CYN was biodegraded at rates from 1.25 to 50 μg/L/day.	[89]
Poland	Biodegradation by *Aeromonas* sp.	CYN biodegradation by Aeromonas sp. was 100% at pH 7 and 25–30 °C but dropped to 0% at pH 11 and colder temperatures.	[90]
New Zealand	Biodegradation by *Sphingomonas* bacteria	*Sphingomonas* species in a ceramic-supported bioreactor removed up to 100% of MCs.	[91]
Methods based on plant coagulants			
Vietnam	Use of *Moringa oleifera * (drumstik tree)	Plant-based coagulants removed up to 97% of turbidity (15 g/L, pH 6.8) and 86–94% at pH 6.2.	[92]
Canada	Use of cactus	*Cactus* species reduced turbidity by 92–98% and lowered pH from 8.89 to 6.	[93]
Morocco	*Opuntia ficus-indica* and *Vicia faba* seed coagulants	At 0.5–1 g/L and pH 5, plant coagulants removed up to 85% of *M. aeruginosa* and about 80% of intracellular cyanotoxins.	[94]
Brazil	Coagulation using *M. oleifera*/flocculation/dissolved air flotation	Combining plant-based methods effectively removed *M. aeruginosa* from water.	[95]
Brazil	*M. oleifera* coagulants	*M. oleifera* coagulants reduced turbidity, suspended matter, and chlorophyll-a by 40–60%.	[96]

## Data Availability

No new data were created or analyzed in this study. Data sharing is not applicable to this article.

## Supplementary Materials

The following supporting information can be downloaded at https://www.mdpi.com/article/10.3390/life15050825/s1. Table S1: Occurrence of cyanotoxins in drinking water systems in Africa; Table S2: Summary data on the occurrence of cyanotoxins in drinking water systems in other regions outside of Africa.
