# Supplementary material for: Global Occurrence of Cyanotoxins in Drinking Water Systems: Recent Advances, Human Health Risks, Mitigation, and Future Directions
Source: Life (Basel). 2025 May 21;15(5):825. doi: 10.3390/life15050825 (PMC12112831; doi:10.3390/life15050825)
Supplement: Supplementary file 1 [file life-15-00825-s001.zip › Table S1.pdf]

Table S1. Occurrence of cyanotoxins in drinking water systems in Africa.

| Country | Type of cyanotoxins | Drinking water system | Key findings and remarks | Reference |
|---|---|---|---|---|
| Mozambique | Microcystins (MCs) (variants: MC-YR, -YR, and –RR) | groundwater, river water | MC-LR, -YR, and -RR were detected in the water samples with concentrations ranging from 6.83 - 7.78 μg $L^{-1}$.( above the WHO maximum limit of 1 μg $L^{-1}$). Ingestion of such water can pose human health risks such as gastroenteritis. | [1] |
| Algeria | Microcystin-LR | Lake Oubeira (source water) | MC concentrations varied between 3 and 29,163 μg $L^{-1}$, depending on season. Highest concentration was recorded in August | [2] |
| Algeria | MC variants | Lake water (source water) | Different variants of MC (with MC-LR dominant) were detected with concentrations ranging from 0.028 to 13.4 μg $L^{-1}$. | [3] |
| Ethiopia | MC variants | drinking water source, Legedadi Reservoir | Six MC variants, namely MC-dmRR, MC-RR, MC-YR, MC-dmLR, MC-LR, and MC-LA, were detected, with concentrations ranging between 61.63 - 453.89 μg $L^{-1}$. The potent MC-LR was the most dominant MCs variant and this could pose extremely high potential public health risk for end-users. | [4] |
| Ethiopia | MC variants | Koka water reservoir | MC variants were detected in the water samples in the following concentrations: MC-LR (815 μg $L^{-1}$), MC-YR (466.6 μg $L^{-1}$), and MC-RR (265.68 μg $L^{-1}$). The highest concentration of the most potent variant, MC-LR, shows the potentially high public health risk posed by its | [5] |

| | | | | |
|---|---|---|---|---|
| | | | presence in drinking water. | |
| Ethiopia | Toxigenic cyanobacteria, MCs | water reservoir (Lake Tana) | Toxigenic cyanobacteria (e.g., *Microcystis aeruginosa*) with the potential to produce microcystins were detected. High levels of microcystins, ranging from 0.58 to 2.65 $\mu$g $L^{-1}$, were detected. | [6] |
| Ethiopia | MCs | water reservoir (Lake Hora-Arsedi) | Considerable concentrations of both intra- and extracellular MCs were detected. Total extracellular MCs concentrations in water of up to 2.99 μg $L^{-1}$ were detected. MC-LR and MC-YR accounted for 71.5 and 28.5%, respectively, of the total MCs measured in the water samples. | [7] |
| Zimbabwe | microcystin-LR | Lake Chivero (source water) | The toxins were produced in cultures of *Microcystis aeruginosa*, with concentrations ranging from 18.02 to 22.48μg $L^{-1}$. These findings raise concerns about the health of people who are drinking the water. | [8] |
| Zimbabwe | microcystins and lipopolysaccharide endotoxins (LE) | source water and treated drinking water | Both toxins were detected in both water systems. LE concentrations in source water ranged between 8 and 3 200 Endotoxin Units (EU) $mL^{-1}$ whilst in treated water ranged from 0.15 to 11 EU $mL^{-1}$. MC concentrations in treated water were below the recommended safe limit for drinking water. | [9] |
| Morocco | MC variants | drinking-water reservoirs | Five MC variants were detected in the cyanobacterial blooms | [10] |

| | | | | |
|---|---|---|---|---|
| | | | (MC-RR, MC-YR, MC-LR, MC-FR, and MC-WR) in the source water, at concentrations as high as 64.4μg $g^{-1}$ dry weight. The results show the importance of regular cyanotoxin monitoring in drinking water systems. | |
| Morocco | MC variants | major water reservoirs (dams) | Nine MC variants namely: (MC-LR); (MC-YR); (MC-LA); (MC-FR); (MC-RF), [Mser7]MC-LR; [Dha7]MC-LR; MC-YAba; and [Mser7]MC-YR, were identified | [11] |
| South Africa | MCs | water reservoir (Hartbeespoort Dam) | Three main MC congeners (MC-RR, -LR and-YR) were isolated from algal samples in the water reservoir. The maximum and minimum concentrations were 268 μg $g^{-1}$ and 0.14 μg $g^{-1}$ DW for MC-RR and MC-YR, respectively. | [12] |
| South Africa | MCs | dam water (water source), water in storage containers, tape water, groundwater | MCs were detected in all the DWS in the following concentrations: tap water (4.3 μg $L^{-1}$), water stored in tanks (4.8 μg $L^{-1}$), and groundwater (0.38 μg $L^{-1}$). The presence of MCs in the water storage containers and tape water suggests that water should be effectively treated before it is used for drinking purposes. | [13] |
| Egypt | microcystins (MC-RR and MC-LR) | source water (Nile River), treated drinking water from an outflow tank | The MCs were detected both in source water and treated water. MCs in outflow tank water were detected in high concentrations (1.1–3.6 μg $L^{-1}$), exceeding the WHO guideline value of 1μg $L^{-1}$ | [14] |

| | | | | |
|---|---|---|---|---|
| | | | for MC-LR in drinking water. These findings showed that MCs could not be completely removed by conventional water treatment processes and so regular monitoring of cyanotoxins in treated water is necessary to provide safe drinking water to consumers | |
| Egypt | cyanobacteria and MCs | domestic water storage reservoirs | The toxic cyanobacterium , *Microcystis aeruginosa* (4.2–5.92× $10^6$ cells per litre), was found in the storage reservoirs. Intra- and extracellular MCs were also detected in reservoir waters at concentrations of 3.5–40 and 1–7.6 μg $L^{-1}$, respectively, exceeding the WHO. The findings indicate that treated-water storage reservoirs should be regularly monitored for the presence of toxins. | [15] |
| Egypt | MCs | surface water (Nile river), groundwater | MCs were found in both water samples, however summertime groundwater concentrations of dissolved MCs were greater (0.1 to 0.84 g $L^{-1}$) than wintertime values (0-0.06 g $L^{-1}$). Accordingly, drinking water from groundwater wells, especially in the summer might be detrimental to health. | [16] |
| Tanzania | MC variants (-LA, -LF, -LR, -LY, -LW, -RR, -YR, -WR, dm MC-RR and dm MC-LR), anatoxin-a, nodularin and | source water (Lake Victoria) and treated pipe water | The cyanotoxins were detected in the source water. Cylindrospermopsin was the most abundant cyanotoxin. MC concentrations ranged from 0.003 to 0.007μg $L^{-1}$ | [17] |

| | | | | |
|---|---|---|---|---|
| | cylindrospermopsin | | for MC-RR, 0.01–0.013μg $L^{-1}$ for MC-LR and 0.004 to 0.01μg $L^{-1}$ for cylindrospermopsin, and 0.01μg $L^{-1}$ for nodularin. No cyanotoxins were detected in treated pipe water samples. The results showed that the water treatment methods were able to remove the cyanotoxins | |
| Ghana | cyanobacterial cells and MCs | drinking water reservoir, treated water | Intracellular MCs (8.73 μg $L^{-1}$), and dissolved toxins were detected in the water reservoir. Cyanobacterial cells (93-3,055 cell $mL^{-1}$), intracellular MCs (0.61 μg $L^{-1}$), and dissolved MCs (0.81 μg $L^{-1}$), were all detected in the final treated water. The results showed that the conventional water treatment methods can not completely remove the cells and their toxins. | [18] |
| Ghana | MCs and cyanobacteria species | drinking water reservoirs | Four cyanobacteria species that produce toxins were identified in the reservoirs and treated water (i.e., *Anabaena flos-aquae, Cylindrospermopsis raciborskii, Microcystis aeruginosa* and *Planktothrix agardhii*). MCs, with the highest concentration (3.21μg $L^{-1}$) were found in the water reservoir. Some water treatment methods are not effective in the removal of all cyanobacterial cells. | [19] |
| Nigeria | MCs | public water supply systems | Dissolved MCs concentrations were higher (>1.0μg $L^{-1}$) than the | [20] |

| | | | | |
|---|---|---|---|---|
| | | | maximum permissible limits for drinking water. These results indicate that there is the need to regularly monitor drinking water systems so as to prevent MCs related human health risks. | |
| Burkina Faso | Cyanobacteria species | drinking water source (Loumbila reservoir) | Thirty toxin-producing species, including microcystin-producing species were found in the water samples. *Microcystis aeruginosa* was the most abundant species. The results point to the presence of various forms of cyanotoxins in the drinking water source. As a result, continuous monitoring of toxins should be done throughout all stages of water treatment, including treated water | [21] |
| South Africa, Botswana, and Zimbabwe | Cyanobacteria species, and cyanotoxins (e.g., cylindrospermopsin, microcystin, nodularin) | Bottom sediment samples from rivers | *Microcystis* spp, *Raphidiopsis raciborskii*, *Phormidium* and *Planktothrix* species were found in the sediments. Cylindrospermopsin polyketide synthetase genes and microcystin/nodularin synthetase genes were amplified from the sediments. The results revealed that the river water contained the cyanotoxins, therefore rural communities using the water for human use may be subject to some adverse health risks. | [22] |
| Kenya | MCs | source water (lake), | 84% of water samples contained MCs with concentration of 3.44μg $L^{-1}$. | [23] |

| | | | | |
|---|---|---|---|---|
| | | household water | The abundance of MCs was in the order MC-RR > MC-YR > MC-LR. | |
| Kenya | MCs | headwater reservoirs | Both toxins were found in water samples. The findings highlighted substantial public health implications, particularly in rural areas, because such reservoirs are important sources of water for human and livestock consumption. | [24] |
| Kenya | MCs | source water (lake) | Low concentrations of MC were detected. Chronic exposure of such water through consumption could pose health risks. | [25] |
| Kenya | MCs, Anatoxin-a | source water (Lake Sonachi and Lake Simbi) | Both cyanotoxins were detected with concentrations of MCs ranging from 1.6 to 39.0μg microcystin-LR equivalents $g^{-1}$DW and Anatoxin-a concentrations ranging from 0 to 2.0μg$g^{-1}$ DW. These findings show that the water needs to be treated to meet WHO guideline levels before consumption | [26] |
| Uganda | MCs and *Microcystis* sp | lake water (Victoria), water treatment plants | Higher cyanobacterial biomass and MCs (ranging from 5 to 10 μg MC-LR equiv. $L^{-1}$) were detected in the water from lake shores. Cell densities and MCs (both intracellular and dissolved) were much reduced to below the values required in drinking water (<1.0 μg MC-LR equiv. $L^{-1}$). | [27] |

**References**

1. Tamele, I.J.; Vasconcelos, V. Microcystin Incidence in the Drinking Water of Mozambique: Challenges for Public Health Protection. *Toxins* **2020**, *12*, 368, doi:10.3390/toxins12060368.
2. Nasri, A.-B.; Bouaïcha, N.; Fastner, J. First Report of a Microcystin-Containing Bloom of the Cyanobacteria Microcystis spp. in Lake Oubeira, Eastern Algeria. *Archives of Environmental Contamination and Toxicology* **2004**, *46*, doi:10.1007/s00244-003-2283-7.
3. Amrani, A.; Nasri, H.; Azzouz, A.; Kadi, Y.; Bouaïcha, N. Variation in Cyanobacterial Hepatotoxin (Microcystin) Content of Water Samples and Two Species of Fishes Collected from a Shallow Lake in Algeria. *Archives of Environmental Contamination and Toxicology* **2014**, *66*, doi:10.1007/s00244-013-9993-2.
4. Habtemariam, H.; Kifle, D.; Leta, S.; Beekman, W.; Lürling, M. Cyanotoxins in drinking water supply reservoir (Legedadi, Central Ethiopia): implications for public health safety. *SN Applied Sciences* **2021**, *3*, 1–10, doi:10.1007/s42452-021-04313-0.
5. Tilahun, S.; Kifle, D.; Zewde, T.W.; Johansen, J.A.; Demissie, T.B.; Hansen, J.H. Temporal dynamics of intra-and extra-cellular microcystins concentrations in Koka reservoir (Ethiopia): Implications for public health risk. *Toxicon* **2019**, *168*, doi:10.1016/j.toxicon.2019.06.217.
6. Mankiewicz-Boczek, J.; Gągała, I.; Jurczak, T.; Urbaniak, M.; Negussie, Y.Z.; Zalewski, M. Incidence of microcystin-producing cyanobacteria in Lake Tana, the largest waterbody in Ethiopia. *African Journal of Ecology* **2015**, *53*, doi:10.1111/aje.12170.
7. Zewde, T.; Kifle, D.; Johansen, J.; Demissie, T.; Hansen, J.; Tadesse, Z. Cyanobacterial abundance and microcystins in water, seston and fish tissues in Lake Hora-Arsedi (Ethiopia). *African Journal of Aquatic Science* **2020**, *45*, 475-485, doi:10.2989/16085914.2020.1723485.
8. Ndebele, M.R.; Magadza, C.H.D. The occurrence of microcystin-LR in Lake Chivero, Zimbabwe. *Lakes & Reservoirs: Research & Management* **2006**, *11*, doi:10.1111/j.1440-1770.2006.00287.x.
9. Mhlanga, L.; Day, J.; Cronberg, G.; Chimbari, M.; Siziba, N.; Annadotter, H. Cyanobacteria and cyanotoxins in the source water from Lake Chivero, Harare, Zimbabwe, and the presence of cyanotoxins in drinking water. *African Journal of Aquatic Science* **2006**, *31*, 165–173, doi:10.2989/16085910609503888.
10. Douma, M.; Ouahid, Y.; Campo, F.F.d.; Loudiki, M.; Mouhri, K.; Oudra, B. Identification and quantification of cyanobacterial toxins (microcystins) in two Moroccan drinking-water reservoirs (Mansour Eddahbi, Almassira). *Environmental Monitoring and Assessment* **2009**, *160*, doi:10.1007/s10661-008-0708-5.
11. Ouhsassi, M.; Khay, E.O.; Laghdach, A.E.; Abdelouahab, F.B.; Ouahrani, A.E.; Idaomar, M.; Abrini, J. Academic Journals - African Journal of Environmental Science and Technology - characterization of cyanobacteria microcystins (cyanotoxins) blooming in the dams of northern morocco: references. *African Journal of Environmental Science and Technology* **2021**, *15*, doi:10.5897/AJEST2020.2967.
12. Mbukwa, E.; Msagati, T.; Mamba, B. Quantitative variations of intracellular microcystin-LR, -RR and -YR in samples collected from four locations in Hartbeespoort Dam in North West Province (South Africa) during the 2010/2011 summer season *International journal of environmental research and public health* **2012**, *9*, doi:10.3390/ijerph9103484.
13. Mokoena, M.M.; Mukhola, M.S. Current Effects of Cyanobacteria Toxin in Water Sources and Containers in the Hartbeespoort Dam Area, South Africa. *International Journal of Environmental Research and Public Health* **2019**, *16*, 4468, doi:10.3390/ijerph16224468.
14. Mohamed, Z.A.; Deyab, M.A.; Abou-Dobara, M.I.; El-Sayed, A.K.; El-Raghi, W.M. Occurrence of cyanobacteria and microcystin toxins in raw and treated waters of the Nile River, Egypt: implication for water treatment and human health. *Environmental Science and Pollution Research* **2015**, *22*, 11716–11727, doi:10.1007/s11356-015-4420-z.
15. Mohamed, Z.A.; Deyab, M.A.; Abou-Dobara, M.I.; El-Raghi, W.M. Occurrence of toxic cyanobacteria and microcystin toxin in domestic water storage reservoirs, Egypt. *Journal of Water Supply: Research and Technology-Aqua* **2016**, *65*, 431–440, doi:10.2166/aqua.2016.115.

16. Mohamed, Z.A.; Alamri, S.; Hashem, M. The link between microcystin levels in groundwater and surface Nile water, and assessing their potential risk to human health. *Journal of Contaminant Hydrology* **2022**, *244*, 103921, doi:10.1016/j.jconhyd.2021.103921.
17. Mchau, G.J.; Machunda, R.; Kimanya, M.; Makule, E.; Gong, Y.Y.; Mpolya, E.; Meneely, J.P.; Elliott, C.T.; Greer, B. First Report of the Co-occurrence of Cylindrospermopsin, Nodularin and Microcystins in the Freshwaters of Lake Victoria, Tanzania. *Exposure and Health* **2020**, *13*, 185–194, doi:10.1007/s12403-020-00372-7.
18. Addico, G.N.D.; Hardege, J.D.; Kohoutek, J.; deGraft-Johnson, K.A.A.; Babica, P. Cyanobacteria and microcystin contamination in untreated and treated drinking water in Ghana. *Advances in Oceanography and Limnology* **2017**, *8*, 92–106, doi:10.4081/aiol.2017.6323.
19. Addico, G.; Hardege, J.; Komarek, J.; Babica, P.; Graft-Johnson, K.d. Cyanobacteria species identified in the Weija and Kpong reservoirs, Ghana, and their implications for drinking water quality with respect to microcystin. *African Journal of Marine Science* **2006**, *28*, 451–-456, doi:10.2989/18142320609504196.
20. Chia, M.; Kwaghe, M. Microcystins contamination of surface water supply sources in Zaria-Nigeria. *Environmental monitoring and assessment* **2015**, *187*, doi:10.1007/s10661-015-4829-3.
21. Ouattara, M.; Zongo, F.; Zongo, B. Species Diversity of Cyanobacteria and Desmids of a Drinking Water Source under Anthropogenic Pressure, and Their Implication in Toxin Production and Water Quality in Sub-Saharan Africa (Burkina Faso, Western Africa). *Journal of Water Resource and Protection* **2021**, *13*, doi:10.4236/jwarp.2021.1312054.
22. Magonono, M.; Oberholster, P.J.; Shonhai, A.; Makumire, S.; Gumbo, J.R. The Presence of Toxic and Non-Toxic Cyanobacteria in the Sediments of the Limpopo River Basin: Implications for Human Health. *Toxins* **2018**, *10*, 269, doi:10.3390/toxins10070269.
23. Otoigo, L. Identification and quantification of Cyanotoxins in Lake and household water around Lake Victoria, Kenya. *AfricArXiv Preprints* **2020**, doi:10.14293/111.000/000007.v1.
24. Mwaura, F.; Koyo, A.O.; Zech, B. Cyanobacterial blooms and the presence of cyanotoxins in small high altitude tropical headwater reservoirs in Kenya. *Journal of Water and Health* **2004**, *2*, 49–57.
25. Krienitz, L.; Dadheech, P.K.; Fastner, J.; Kotut, K. The rise of potentially toxin producing cyanobacteria in Lake Naivasha, Great African Rift Valley, Kenya. *Harmful Algae* **2013**, *27*, doi:10.1016/j.hal.2013.04.005.
26. Ballot, A.; Krienitz, L.; Kotut, K.; Wiegand, C.; Pflugmacher, S. Cyanobacteria and cyanobacterial toxins in the alkaline crater lakes Sonachi and Simbi, Kenya. *Harmful Algae* **2005**, *4*, 139–150, doi:10.1016/j.hal.2004.01.001.
27. Olokotum, M.; Humbert, J.-F.; Quiblier, C.; Okello, W.; Semyalo, R.; Troussellier, M.; Marie, B.; Baumann, K.; Kurmayer, R.; Bernard, C. Characterization of Potential Threats from Cyanobacterial Toxins in Lake Victoria Embayments and during Water Treatment. *Toxins* **2022**, *14*, 664, doi:10.3390/toxins14100664.
