# Supplementary material for: Global Occurrence of Cyanotoxins in Drinking Water Systems: Recent Advances, Human Health Risks, Mitigation, and Future Directions
Source: Life (Basel). 2025 May 21;15(5):825. doi: 10.3390/life15050825 (PMC12112831; doi:10.3390/life15050825)
Supplement: Supplementary file 1 [file life-15-00825-s001.zip › Table S2.pdf]

Table S2. Summary data on occurrence of cyanotoxins in drinking water systems in other regions outside Africa.

Location	Years of study	Species	Cyanotoxins	MC Concentration in the water (µg L^{-1})	MC Concentration in cells (mg g DW^{-1})	Reference
**A: ASIA**						
Lake Dong Ting, China	NA	*Microcystis* spp.	MC	NA		[1]
Lake Taihu, China	2004	*Microcystis* spp.	MC	34 200	0.59	[2]
Lake Taihu, China	2005, 2006	*Microcystis* spp.	MC	3.56	1.81	[3]
Lake Taihu, China	2007	*Microcystis* spp.	Not mentioned	NA		[4]
Lake Chao, China	2007	*Microcystis aeruginosa*	MC-RR	NA	4.799	[5]
Guangting Reservoir, China	2008	*Microcystis* ssp.	MC-RR, MC-LR	1.15	0.41	[6]
Huai River, China	2009	*Microcystis* spp.	MC-LR, MC-RR, MC-YR	1.846		[7]
Lake Dianchi, China	2009, 2010	*Microcystis* spp.	MC-LR	1.03		[8]
Lake Erhai, China	2010	*Microcystis* spp.	MC-RR, MC-LR	8.95		[9]
Yanghe Reservoir, China	2010	*Microcystis* spp.	MC	2.12		[10]
Lake Taihu, China	2010	*Microcystis* spp.	MC-LR	44		[11]
Lake Taihu, China	2012, 2013, 2014	*Microcystis flos-aquae, M. aeruginosa, M. wesenbergii, M. viridis and M. ichthyoblabe*	MC-RR, MC-LR	NA	1.1411, 1.664, 1.3868 respectively	[12]
Lake Taihu, China	2013, 2014	Cyanobacteria	MC-LR, MC-RR, MC-YR	NA	2.37	[13]

Lake Chaohu, China	2013, 2014	*Microcystis. aeruginosa*	MC	1.07	[14]
Dashahe Reservoir, China	2015	*Microcystis* spp.	MC-LR, MC-RR, MC-YR	453.9	[15]
Manjalar Dam, India	NA	*Microcystis. aeruginosa*	MC-LR	NA	[16]
Dal Lake, India	2018 - 2020	*Microcystis* spp., *Dolichospermum* sp.	Not mentioned	NA	[17]
Biwa Lake, Japan	1998	*Raphidiopsis mediterranea, Aphanizomenon issatschenkoi, Microcustis* sp., *Anabaena* sp.	HTX	NA	[18]
Kovada Lake, Turkey	2005	*Microcystis aeruginosa, Synechococcus sp., Phormidium limosum, P. Formosa, Planktothrix limnetica*	MC-LR, MC-RR, MC-LA, MC-LW, MC-LF	98.9	[19]
Yezin Dam, Myanmar	2020	*R. raciborskii, Microcystis* sp.	CYN, MC	0.12 and 0.34 respectively	[20]
**B: AUSTRALIA**					
Solomon Dam, Australia	1979	*Cylindrospermopsis raciborskii*	CYN	NA	[21]
Lake Alexandrina, Australia	1994 - 1995	*Nodularia spumigena*	NOD	NA	[22]
Lake Albert, Australia	1994 - 1995	*Nodularia spumigena*	NOD	NA	[22]
Shallow lakes, Queensland, Australia	1997 - 1999	*Cylindrospermopsis raciborskii, Limnothrix aff. redekei, Pseudanabaena*	CYN	18.9	[23]

		limnetica, Planktolyngbya subtilis				
Myponga Reservoi, Australia	2000	*Phormidium* aff. *formosum*	Unknown	NA		[24]
Upper Paskeville Reservoir, Australia	2000	*Phormidium* aff. *amoenum*	Unknown	NA		[24]
Murray River and its tributaries, Australia	2009	*Anabaena circinalis, Microcystis flos-aquae, Cylindrospermopsis raciborskii,*	STX, CYN	NA		[25]
Waikato River, New Zealand	2003	*Cylindrospermopsis raciborskii*	CYN, STX	NA		[26]
Lake Waahi, New Zealand	2003	*Cylindrospermopsis raciborskii*	CYN, do-CYN	NA		[27]
**C: EUROPE**						
Torrão Reservoir, Portugal	1992	*Microcystis Aeruginosa, M. wesenbergii*	MC	NA	1.6	[28]
Aguieira Reservoir, Portugal	1992	*Microcystis aeruginosa*	MC-LR, MC-HilR	NA	5.6	[28]
Sulejów Dam, Poland	1993, 1994, 1995, 1996, 1997, 1998	*Microcystis* spp.	MC-LR	no data, no data, 6.8, 5.3, 4.6, 2.7 respectively	0.149, 0.191, 0.427, 0.227, 0.286, 0.168 respectively	[29]
Jeziorsko Dam, Poland	1996, 1997, 1998	*Microcystis aeruginosa, Aphanizomenon flos-aquae*	MC-RR, MC-YR, MC-LR	NA	0.912, 1.069, 1.347, 0.316 respectively	[30]

	, 2000					
Jeziorsko Dam, Poland	1997	*Microcystis aeruginosa*	MC	NA	2.155	[31]
Sulejów Dam, Poland	1996, 1997, 1998, 2000, 2001	*Microcystis aeruginosa*	MC-RR, MC-YR, MC-LR	NA	1.687, 0.562, 1.261, 1.292 respectively	[30]
Sulejów Dam, Poland	1998, 1999	*Microcystis aeruginosa*	MC	NA	1.511, 1.437 respectively	[31]
Sulejów Dam, Poland	1999	*Microcystis* ssp.	MC	0.8	0.86	[32]
Siemianówka Dam, Poland	1999	*Microcystis aeruginosa*	MC-RR, MC-YR, MC-LR	NA	0.439	[30]
Sulejów Dam, Poland	2003, 2004	*Microcystis* ssp.	MC	2.86, 5.83 respectively		[33]
Sulejów Dam, Poland	2004	*Microcystis* ssp.	MC	4.67		[34]
Sulejów Dam, Poland	2005	*Microcystis aeruginosa*	MC	2.83		[35]
Siemianówka Dam, Poland	2005, 2006	*Microcystis* ssp.	MC-LR	11.9, 173.8 respectively	0.971, 7.827 respectively	[36]
Jeziorsko Dam, Poland	2008	*Microcystis Aeruginosa, Aphanizomenon* sp.	MC-RR, MC-YR, MC-LR	1.77		[37]
Siemianówka Dam, Poland	2008	*Microcystis* ssp.	MC-RR, MC-YR	4.6	0.069	[38]
Siemianówka Dam, Poland	2009	*Planktothrix agardhii*	MC-RR, MC-RY, MC-VR, MC-LR, MC-YR	10		[39]

Obrzyca River, Poland	2019	*Microcystis aeruginosa*	MC-RR, dmMC-LR, MC-LF, MC-LR,MC-LY, MC-LW	0.83	[40]
Saint-Caprais Reservoir, France	1998 - 1999	*Aphanizomenon flos-aquae*	MC-LR	0.052	[41]
Inniscarra Reservoir, Ireland	NA	*Planktothrix formosa*	HTX	34	[42]
Santillana Reservoir, Spain	2002	*Microcystis aeruginosa, Microcystis flos-aquae, Aphanizomenon flos-aquae*	MC-LR, MC-YR, MC-RR	55	[43]
Atazar Reservoir, Spain	2003	*Planktothrix rubescens*	MC-LR, MC-RR, MC-YR, MC-YA, MC-HtyR	19.1	[44]
Cogotas Reservoir, Spain	2006	*Microcystis flos-aquae, Aphanizomenon* sp., *Pseudanabaena limnetica, Planktothrix agardhii*	MC-LR, MC-YR, MC-RR	64.8	[45]
Encinarejo Reservoir, Spain	2008	*Chrysosporum ovalisporum, Dolichospermum* sp.	CYN	3.4	[46]
San Juan Dam, Spain	2012	*Microcystis aeruginosa, M. viridis, M. wesenbergii, Aphanizomenon flos-aquae, Dolichospermum crassum,*	MC-LR, MC-YR, MC-RR	49.5	[47]

		Woronichinia naegeliana			
Ćelije Reservoir, Serbia	2004	*A. circinalis, Aph. flos-aquae, M. aeruginosa*	MC-LR	650	[48]
Borovitsa Dam, Bulgaria	2006	*Cuspidothrix* sp.	ATX	NA	[49]
Borovitsa Dam, Bulgaria	2006	*Aphanizomenon flos-aquae, Microcystis pulverea* and *Synechococcus elongatus* respectively	MC, NOD	0.12, 0.18 respectivel y	[50]
Trakiets Reservoir, Bulgaria	2008 , 2009	NA	MC	0.9, 0.014 respectivel y	[51]
Studena Reservoir, Bulgaria	2011	Unknown	MC	0.1	[52]
Studena Reservoir, Bulgaria	2013	Unknown	MC-LR, MC-RR, MC-YR	10.9	[53]
Studena Reservoir, Bulgaria	2015	NA	MC	0.6	[54]
Lake Marathonas , Greece	2010	*Microcystis* sp.	MC-YR, MC-LR, MC-RR	0.717, 0.451, 0.174 respectivel y	[55]
Boskovice Reservoir, Czech Republic	2010	*Microcystis* sp.	MC	0.13	[56]
Fryšták Reservoir, Czech Republic	2010	*Microcystis* sp., *Phormidium* sp., *Chroococcus* sp.	MC	2.05	[56]
Husinec Reservoir, Czech Republic	2010	*Dolichospermum* sp.	MC	1.77	[56]
Mariánské lázně Reservoir,	2010	*Dolichospermum* sp., *Microcystis* sp.	MC	0.46	[56]

Czech Republic					
Mostiště Reservoir, Czech Republic	2010	*Dolichospermum* sp., *Microcystis* sp.	MC	3.83	[56]
Římov Reservoir, Czech Republic	2010	*Microcystis* sp.	MC	0.15	[56]
Vír Reservoir, Czech Republic	2010	*Microcystis* sp.	MC	14.3	[56]
Vranov Reservoir, Czech Republic	2010	*Microcysis* sp., *Dolichorspermum* sp.	MC	4.19	[56]
Lough Neagh, Ireland	2008	*Aphanizomenon flos-aquae*	MC	NA	[57]
Weida Reservoir, Germany	2000, 2001	*Planktothrix rubescens*	MC-RR	NA	[58]
Lake Trasimeno, Italy	2000, 2005	Not mentioned	MC-RR, MC-YR, MC-LR, MC-LA	NA	[59]
Lake Vico, Italy	2006	*Planktothrix rubescens*	MC-RR, MC-LR	NA	[59]
Occhito Lake, Italy	2010	*Planktothrix rubescens*	MC-HtyR	0.025	[60]
Lake Alto Flumendosa, Italy	2011, 2012, 2013	*Planktothrix rubescens, Woronichinia naegeliana, Microcystis botrys, Dolichospermum planctonicum*	MC	54.94, 75.526, 8.755 respectively	[61]
Lake Immeln, Sweden	2012	NA	MC-LR, MC-RR,	814.96	[62]
Lake Mälaren, Sweden	2013	NA	HTX, MC (MC-LR, MC-RR)	10.6 and 4.54 respectively	[62]

Lake Långasjön, Sweden	2013	NA	MC-LR, MC-LF	7,47	[62]
Lake Sydvatten, Sweden	2013	NA	MC-RR, ATX	1.78 and 1.3 respectively	[62]
Infiltration pond, Sweden	2013	NA	MC-LR, MC-RR	1.55	[62]
Lake Űlmiste, Sweden	2014	NA	MC-LR, MC-RR, MC-YR, MC-HphR	35,87	[62]
Gorky Reservoir, Russia	2013	*Aphanizomenon flos-aquae, Dolichospermum lemmermannii, M. aeruginosa*	STX	26	[63]
Novosibirsk Reservoir, Russia	2013	*Aphanizomenon flos-aquae, M. aeruginosa*	STX	2.3	[63]
Ivankovo Reservoir, Russia	2016, 2018	Not mentioned	MC	NA	[64]
Uglich Reservoir, Russia	2016, 2018	Not mentioned	MC	NA	[64]
Rybinsk Reservoir, Russia	2016, 2018	Not mentioned	MC	NA	[64]
Gorky Reservoir, Russia	2016, 2018	Not mentioned	MC	NA	[64]
Cheboksary Reservoir, Russia	2016, 2018	Not mentioned	MC	NA	[64]
Kuybyshev Reservoir, Russia	2016, 2018	Not mentioned	MC	NA	[64]
Saratov Reservoir, Russia	2016, 2018	Not mentioned	MC	NA	[64]
Volgograd Reservoir, Russia	2016, 2018	Not mentioned	MC	NA	[64]

Nizhnekamsk Reservoir, Russia	2016, 2018	Not mentioned	MC	NA		[64]
Votkinsk Reservoir, Russia	2016, 2018	Not mentioned	MC	NA		[64]
Kamsk Reservoir, Russia	2016, 2018	Not mentioned	MC	NA		[64]
Tsimlyansk Reservoir, Russia	2016, 2018	Not mentioned	MC	NA		[64]
**D: SOUTH AMERICA**						
Tabocas reservoir, Brazil	1996	*Microcystis* ssp.	MC	19.5		[65]
Lake Bolonha, Brazil	1999	*Radiocystis fernandoi*	MC-LR	NA	3.83	[66]
Utinga Reservoir, Brazil	1999-2000	*Microcystis viridis, Radiocystis fernandoi*	MC	4.22, 2.47 respectively		[67]
Faxinal Reservoir, Brazil	2004	*Anabaena crassa*	ATX	NA		[68]
Alagoinha Reservoir, Brazil	2009	Not mentioned	MC, STX, Neo-STX	13542, 124.51, 71.35 respectively		[69]
Arcoverde Reservoir, Brazil	2005	Not mentioned	MC	687.5		[69]
Carpina Reservoir, Brazil	2006, 2009, 2011	Not mentioned	MC, CYN, STX, Neo-STX	303053.9, 0.72, 299.35, 1552.6 respectively		[69]
Duas Unas Reservoir, Brazil	2006, 2009	Not mentioned	MC, CYN, STX, Neo-STX	122053.9, 143.0, 1.94, 140.4 respectively		[69]

Ingazeira Reservoir, Brazil	2009	Not mentioned	MC, STX, Neo-STX	3399.7, 299.73, 1045.8 respectively	[69]
Ipojuca Reservoir, Brazil	2010	Not mentioned	MC, CYN, STX, Neo-STX	14.01, 201.5, 195.42, 344.1 respectively	[69]
Jazigo Reservoir, Brazil	2005	Not mentioned	MC, STX, Neo-STX	16.7, 1.47, 0.74 respectively	[69]
Jucazinho Reservoir, Brazil	2004, 2009	Not mentioned	MC, CYN, STX, Neo-STX, ATX	489.2, 2718.0, 316.06, 69.57 respectively	[69]
Mundaú Reservoir, Brazil	2008, 2009	Not mentioned	MC, STX, Neo-STX	16858.4, 7.76, 145.2 respectively	[69]
Tapacurá Reservoir, Brazil	2006, 2009	Not mentioned	MC, STX, Neo-STX, ATX	1063.6, 173.79, 309.62, 13.15 respectively	[69]
Venturosa Reservoir, Brazil	2009	Not mentioned	MC, STX, Neo-STX	17.3, 0.42, 1.34 respectively	[69]
Ingazeira Reservoir, Brazil	2009	*Cylindrospermopsis raciborskii, Sphaerospermopsis aphanizomenoides, Planktothrix zahidii*	MC, STX	NA	[69]
Mundaú Reservoir, Brazil	2009	*M. aeruginosa, M. flos-aquae*	MC	NA	[69]

Argemiro de Figueiredo Reservoir, Brazil	2007-2009	*Pseudanabaena limnetica, C. raciborskii, Aphanocapsa incerta, Dolichospermum circinalis, Trichodesmium lacustre, M. aeruginosa, Microcystis* sp.	MC-LR	13.8	[70]
Alagados Reservoi, Brazil	2015-2016	*C. raciborskii*	STX	0.0221	[71]
Amazon River, Brazil	2015	*Limnothrix planctonica*	MC-LR	2.1	[72]
Billings Reservoir, Brazil	2017	*R. raciborskii, Microcystis* ssp.	MC	21.41	[73]
Billings Reservoir, Brazil	2018	*R. raciborskii, Microcystis* ssp.	MC, STX	5.08, 0.05 respectively	[73]
Billings Reservoir, Brazil	2019	*R. raciborskii, Microcystis* ssp.	MC, STX	56.88, 0.13 respectively	[73]
Itupararanga Reservoir, Brazil	2017-2018	*Raphidiopsis raciborskii*	STX, MC	0.23, 0.14 respectively	[74]
San Roque Reservoir, Argentina	1997-2011	Not mentioned	MC-LR, MC-YR, MC-RR, NOD	NA	[75]
San Roque Reservoir, Argentina	1999, 2000, 2001	Not mentioned	MC	1.42, 11.74, 18.39 respectively	[76]
San Roque Reservoir, Agrentina	2006-2007	*Microcystis* sp., *Pseudoanabaena* sp., *Anabaena* sp.	MC (MC-LR, MC-RR, MC-RR, MC-YR), ATX	9.21, 2.7 respectively	[77]
Paso de las Piedras Dam, Argentina	2000	*Microcystis* ssp.	MC	0.17	Conti, 2005 #516}

Río de la Plata Estuary, Argentina	2005	*Microcystis aeruginosa*	MC-LR	8.6	[78]
Paraná River, Argentina	2005	*Microcystis aeruginosa*	MC-LR, MC-RR	0.13	[79]
Paraná River, Argentina	2006	*Microcystis aeruginosa*	MC-LR, MC-RR	39.13	[79]
Paraná River, Argentina	2007	*Microcystis aeruginosa*	MC-LR	9.7	[79]
Paraná River, Argentina	2008	*Microcystis aeruginosa*	MC-LR	18.9	[79]
Laguna Lo Galindo, Chile	2013	*Microcystis* sp.	MC-LR, MC-RR, MC-YR, MC-LA	115.4	[80]
Abreo-Malpaso Reservoir, Colombia	2015 - 2016	*Microcystis aeruginosa* *Aphanocapsa* sp., *Radiocystis* sp. *Woronichinia* sp.	MC (MC-LR, MC-YR, MC-RR, MC-LF, MC-LW), NOD, CYN	43.6, 3.468 0.047 respectivel y	[81]
Pañe Reservoir, Peru	2017	*Dolichospermum circinale*	ATX, MC	0.45, 5.2 respectivel y	[82]
**E: NORTH AMERICA**					
Lake Coal, Canada	1990 - 1992	*Microcystis aeruginosa, Aphanizomenon flos-aquae, Anabaena* spp., *Gomphosphaeria* spp.	MC-LR	NA	[83]
Lake Driedmeat, Canada	1990 - 1992	*Microcystis aeruginosa, Aphanizomenon flos-aquae, Anabaena* spp., *Gomphosphaeria* spp.	MC-LR	0.003	[83]

Lake Little Beaver, Canada	1990 - 1992	*Microcystis aeruginosa, Aphanizomenon flos-aquae, Anabaena* spp., *Gomphosphaeria* spp.	MC-LR	0.005		[83]
Lake of the Woods, Canada	2004	*Aphanizomenon. flos-aquae, Anabaena flos-aquae, A. lemmermannii, A. mendotae, Aphanothece* spp., *Pseudanabaena* spp., *Woronichinia* spp.	MC-LR		115	[84]
Lake Mathews, USA	2003	Not mentioned	MC		32.4	[85]
Diamond Valley Lake, USA	2004	Not mentioned	MC		288.0	[85]
Lake Perris, USA	2004	Not mentioned	MC		15.6	[85]
Lake Erie, USA	2014	*Microcystis ssp.*	MC	210		[86]
Water treatment plant in Ohio, USA	2017	*Microcystis ssp.*	MC	NA		[87]
Sacramento –San Joaquin River Delta in California, USA	2017	*Microcystis* ssp.	MC-LR		0.162	[88]
Lake Manatee, USA	2019	NA	MC, NOD	0.47		[89]
Lake Washington, USA	2019	NA	MC, NOD	0.28		[89]

Los Berros water treatment plant, Mexico	2006	*Cylindrospermopsis* sp., *Anabaena* sp., *Aphanizomenon* sp.	ATX, CYN	129.3, 6.9 respectively	[90]
Valle de Bravo Reservoir, Mexico	2017	*Microcystis aeruginosa, Microcystis wesenbergii, Woronichinia naegeliana*	MC	9.6	[91]
Lake Bosque Azul, Mexico	2017	*Limnothrix* sp., *Raphidiopsis* sp., *Planktothrix* sp.	MC-LR	5.3	[92]
Lake La Encantada, Mexico	2017	*Limnothrix* sp., *Raphidiopsis* sp., *Planktothrix* sp.	MC-LR	3.1	[92]
Lake San Lorenzo, Mexico	2017	*Limnothrix* sp., *Raphidiopsis* sp., *Planktothrix* sp.	MC-LR	3.8	[92]
Valle de Bravo Dam, Mexico	2019	*M. smithii, M. aeruginosa, M. viridis M. flos-aquae, Aphanocapsa planctonica, and Dolichospermum crassum,*	MC	71	[93]

References

1. Feng, H.; Clara, T.; Huang, F.; Wei, J.; Yang, F. Identification and characterization of the dominant Microcystis sp. cyanobacteria detected in Lake Dong Ting, China. *Journal of Toxicology and Environmental Health, Part A* **2019**, doi:10.1080/15287394.2019.1700604.
2. Liu, Y.; Chen, W.; Li, D.; Huang, Z.; Shen, Y.; Liu, Y. Cyanobacteria-/cyanotoxin-contaminations and eutrophication status before Wuxi Drinking Water Crisis in Lake Taihu, China. *Journal of Environmental Sciences* **2011**, *23*, doi:10.1016/S1001-0742(10)60450-0.
3. Song, L.; Chen, W.; Peng, L.; Wan, N.; Gan, N.; Zhang, X. Distribution and bioaccumulation of microcystins in water columns: A systematic investigation into the environmental fate and the risks associated with microcystins in Meiliang Bay, Lake Taihu. *Water Research* **2007**, *41*, doi:10.1016/j.watres.2007.02.013.
4. Qin, B.; Zhu, G.; Gao, G.; Zhang, Y.; Li, W.; Paerl, H.W.; Carmichael, W.W. A Drinking Water Crisis in Lake Taihu, China: Linkage to Climatic Variability and Lake Management. *Environmental Management* **2009**, *45*, doi:10.1007/s00267-009-9393-6.

5. Krüger, T.; Wiegand, C.; Kun, L.; Luckas, B.; Pflugmacher, S. More and more toxins around–analysis of cyanobacterial strains isolated from Lake Chao (Anhui Province, China). *Toxicon* **2010**, *56*, doi:10.1016/j.toxicon.2010.09.004.
6. Dai, R.; Liu, H.; Qu, J.; Ru, J.; Hou, Y. Cyanobacteria and their toxins in Guanting Reservoir of Beijing, China. *Journal of Hazardous Materials* **2008**, *153*, doi:10.1016/j.jhazmat.2007.08.078.
7. Tian, D.; Zheng, W.; Wei, X.; Sun, X.; Liu, L.; Chen, X.; Zhang, H.; Zhou, Y.; Chen, H.; Zhang, H.; et al. Dissolved microcystins in surface and ground waters in regions with high cancer incidence in the Huai River Basin of China. *Chemosphere* **2013**, *91*, doi:10.1016/j.chemosphere.2013.01.051.
8. Wu, Y.; Li, L.; Gan, N.; Zheng, L.; Ma, H.; Shan, K.; Liu, J.; Xiao, B.; Song, L. Seasonal dynamics of water bloom-forming Microcystis morphospecies and the associated extracellular microcystin concentrations in large, shallow, eutrophic Dianchi Lake. *Journal of Environmental Sciences* **2014**, *26*, doi:10.1016/j.jes.2014.06.031.
9. Yu, G.; Jiang, Y.; Song, G.; Tan, W.; Zhu, M.; Li, R. Variation of Microcystis and microcystins coupling nitrogen and phosphorus nutrients in Lake Erhai, a drinking-water source in Southwest Plateau, China. *Environmental Science and Pollution Research* **2014**, *21*, doi:10.1007/s11356-014-2937-1.
10. Wang, L.; Liu, L.; Zheng, B. Eutrophication development and its key regulating factors in a water-supply reservoir in North China. *Journal of Environmental Sciences* **2013**, *25*, doi:10.1016/S1001-0742(12)60120-X.
11. Sakai, H.; Hao, A.; Iseri, Y.; Wang, S.; Kuba, T.; Zhang, Z.; Katayama, H. Occurrence and distribution of microcystins in Lake Taihu, China. *The Scientific World Journal* **2013**, *2013*, doi:10.1155/2013/838176.
12. Hu, L.; Shan, K.; Lin, L.; Shen, W.; Huang, L.; Gan, N.; Song, L. Multi-Year Assessment of Toxic Genotypes and Microcystin Concentration in Northern Lake Taihu, China. *Toxins 2016, Vol. 8, Page 23* **2016**, *8*, doi:10.3390/toxins8010023.
13. Xue, Q.; Steinman, A.D.; Xie, L.; Yao, L.; Su, X.; Cao, Q.; Zhao, Y.; Cai, Y. Seasonal variation and potential risk assessment of microcystins in the sediments of Lake Taihu, China. *Environmental Pollution* **2020**, *259*, doi:10.1016/j.envpol.2019.113884.
14. Yang, Z.; Kong, F.; Zhang, M. Groundwater contamination by microcystin from toxic cyanobacteria blooms in Lake Chaohu, China. *Environmental Monitoring and Assessment 2016 188:5* **2016**, *188*, 1–9, doi:10.1007/s10661-016-5289-0.
15. Xiang, L.; Li, Y.-W.; Liu, B.-L.; Zhao, H.-M.; Li, H.; Cai, Q.-Y.; Mo, C.-H.; Wong, M.-H.; Li, Q.X. High ecological and human health risks from microcystins in vegetable fields in southern China. *Environment International* **2019**, *133*, doi:10.1016/j.envint.2019.105142.
16. Anahas, A.M.P.; Gayathri, M.; Muralitharan, G. Isolation and Characterization of Microcystin-Producing Microcystis aeruginosa MBDU 626 from a Freshwater Bloom Sample in Tamil Nadu, South India. **2013**, doi:10.1007/978-81-322-1087-0_16.
17. Bashir, F.; Bashir, A.; Rajput, V.D.; Bouaïcha, N.; Fazili, K.M.; Adhikari, S.; Negi, Y.; Minkina, T.; Almalki, W.H.; Ganai, B.A. Microcystis sp. AE03 strain in Dal Lake harbors cylindrospermopsin and microcystin synthetase gene cluster. *Frontiers in Sustainable Food Systems* **2022**, *6*, 1036111, doi:10.3389/fsufs.2022.1036111.
18. Watanabe, M.F.; Tsujimura, S.; Oishi, S.; Niki, T.; Namikoshi, M. Isolation and identification of homoanatoxin-a from a toxic strain of the cyanobacterium Raphidiopsis mediterranea Skuja isolated from Lake Biwa, Japan. *Phycologia* **2003**, *42*, 364–369, doi:10.2216/i0031-8884-42-4-364.1.
19. Gurbuz, F.; Metcalf, J.S.; Karahan, A.G.; Codd, G.A. Analysis of dissolved microcystins in surface water samples from Kovada Lake, Turkey. *Science of The Total Environment* **2009**, *407*, 4038–4046, doi:10.1016/j.scitotenv.2009.02.039.
20. Swe, T.; Miles, C.O.; Cerasino, L.; Mjelde, M.; Kleiven, S.; Ballot, A. Microcystis, Raphidiopsis raciborskii and Dolichospermum smithii, toxin producing and non-toxigenic cyanobacteria in Yezin Dam, Myanmar. *Limnologica* **2021**, *90*, 125901, doi:10.1016/j.limno.2021.125901.

21. Bourke, A.T.C.; Hawes, R.B.; Neilson, A.; Stallman, N.D. An outbreak of hepato-enteritis (the Palm Island mystery disease) possibly caused by algal intoxication. *Toxicon* **1983**, *21*, doi:10.1016/0041-0101(83)90151-4.
22. Fitzgerald, D.J.; Cunliffe, D.A.; Burch, M.D. Development of health alerts for cyanobacteria and related toxins in drinking water in South Australia. *Environmental Toxicology* **1999**, *14*, doi:10.1002/(SICI)1522-7278(199902)14:1<203::AID-TOX26>3.0.CO;2-X.
23. McGregor, G.B.; Fabbro, L.D. Dominance of Cylindrospermopsis raciborskii (Nostocales, Cyanoprokaryota) in Queensland tropical and subtropical reservoirs: Implications for monitoring and management. *Lakes & Reservoirs: Research & Management* **2000**, *5*, doi:10.1046/j.1440-1770.2000.00115.x.
24. Baker, P.D.; Steffensen, D.A.; Humpage, A.R.; Nicholson, B.C.; Falconer, I.R.; Lanthois, B.; Fergusson, K.M.; Saint, C.P. Preliminary evidence of toxicity associated with the benthic cyanobacterium Phormidium in South Australia. *Environmental Toxicology* **2001**, *16*, doi:10.1002/tox.10009.
25. Al-Tebrineh, J.; Merrick, C.; Ryan, D.; Humpage, A.; Bowling, L.; Neilan, B.A. Community Composition, Toxigenicity, and Environmental Conditions during a Cyanobacterial Bloom Occurring along 1,100 Kilometers of the Murray River. *Applied and Environmental Microbiology* **2012**, *78*, 263–272, doi:10.1128/AEM.05587-11.
26. Ryan, E.F.; Hamilton, D.P.; Barnes, G.E. Recent occurrence of Cylindrospermopsis raciborskii in Waikato lakes of New Zealand. *New Zealand Journal of Marine and Freshwater Research* **2003**, *37*, 829–836, doi:10.1080/00288330.2003.9517212.
27. Wood, S.A.; Stirling, D.J. First identification of the cylindrospermopsin-producing cyanobacterium Cylindrospermopsis raciborskii in New Zealand. *New Zealand Journal of Marine and Freshwater Research* **2003**, *37*, 821–828, doi:10.1080/00288330.2003.9517211.
28. Vasconcelos, V.M.; Sivonen, K.; Evans, W.R.; Carmichael, W.W.; Namikoshi, M. Hepatotoxic microcystin diversity in cyanobacterial blooms collected in portuguese freshwaters. *Water Research* **1996**, *30*, doi:10.1016/0043-1354(96)00152-2.
29. Kabziński, A.K.M.; Juszczak, R.; Miękoś, E.; Tarczynska, E.; Sivonen, K.; Rapala, J. The First Report about the Presence of Cyanobacterial Toxins in Polish Lakes. *Polish Journal of Environmental Studies* **2000**, *9*, 171–178.
30. Jurczak, T.; Tarczyńska, M.; Karlsson, K.; et al. Characterization and diversity of cyanobacterial hepatotoxins (Microcystins) in blooms from polish freshwaters identified by liquid chromatography-electrospray ionisation mass spectrometry. *Chromatographia* **2004**, *59*, 571–578, doi:https://doi.org/10.1365/s10337-004-0279-8.
31. Mankiewicz, J.; Walter, Z.; Tarczynska, M.; Palyvoda, O.; Wojtysiak-Staniaszczyk, M.; Zalewski, M. Genotoxicity of cyanobacterial extracts containing microcystins from Polish water reservoirs as determined by SOS chromotest and comet assay. *Environmental Toxicology* **2002**, *17*, doi:10.1002/tox.10061.
32. Tarczyńska, M.; Romanowska-Duda, Z.; Jurczak, T.; Zalewski, M. Toxic cyanobacterial blooms in a drinking water reservoir - causes, consequences and management strategy. *Water Supply* **2001**, *1*, doi:10.2166/ws.2001.0043.
33. Izydorczyk, K.; Jurczak, T.; Wojtal-Frankiewicz, A.; Skowron, A.; Mankiewicz-Boczek, J.; Tarczyńska, M. Influence of abiotic and biotic factors on microcystin content in Microcystis aeruginosa cells in a eutrophic temperate reservoir. *Journal of Plankton Research* **2008**, *30*, doi:10.1093/plankt/fbn006.
34. Mankiewicz-Boczek, J.; Izydorczyk, K.; Romanowska-Duda, Z.; Jurczak, T.; Stefaniak, K.; Kokocinski, M. Detection and monitoring toxigenicity of cyanobacteria by application of molecular methods. *Environmental Toxicology* **2006**, *21*, doi:10.1002/tox.20200.
35. Gągała, I.; Izydorczyk, K.; Skowron, A.; Kamecka-Plaskota, D.; Stefaniak, K.; Kokociński, M.; Mankiewicz-Boczek, J. Appearance of toxigenic cyanobacteria in two Polish lakes dominated by Microcystis aeruginosa and Planktothrix agardhii and environmental factors influence. *Ecohydrology & Hydrobiology* **2010**, *10*, doi:10.2478/v10104-009-0045-5.

36. Grabowska, M.; Pawlik-Skowrońska, B. Replacement of chroococcales and nostocales by oscillatoriales caused a significant increase in microcystin concentrations in a dam reservoir. *Oceanological and Hydrobiological Studies* **2009**, *37*, 23–33, doi:10.2478/v10009-008-0016-y.
37. Mankiewicz-Boczek, J.; Palus, J.; Gągała, I.; Izydorczyk, K.; Jurczak, T.; Dziubałtowska, E.; Stępnik, M.; Arkusz, J.; Komorowska, M.; Skowron, A.; et al. Effects of microcystins-containing cyanobacteria from a temperate ecosystem on human lymphocytes culture and their potential for adverse human health effects. *Harmful Algae* **2011**, *10*, 356–365, doi:10.1016/j.hal.2011.01.001.
38. Grabowska, M.; Mazur-Marzec, H. The effect of cyanobacterial blooms in the Siemianówka Dam Reservoir on the phytoplankton structure in the Narew River. *Oceanological and Hydrobiological Studies* **2011**, *40*, 19–26, doi:10.2478/s13545-011-0003-x.
39. Kobos, J.; Błaszczyk, A.; Hohlfeld, N.; Toruńska-Sitarz, A.; Krakowiak, A.; Hebel, A.; Sutryk, K.; Grabowska, M.; Toporowska, M.; Kokociński, M.; et al. Cyanobacteria and cyanotoxins in Polish freshwater bodies. *Oceanological and Hydrobiological Studies* **2014**, *42*, 358–378, doi:10.2478/s13545-013-0093-8.
40. Czyżewska, W.; Piontek, M.; Łuszczyńska, K. The Occurrence of Potential Harmful Cyanobacteria and Cyanotoxins in the Obrzyca River (Poland), a Source of Drinking Water. *Toxins* **2020**, *12*, 284, doi:10.3390/toxins12050284.
41. Maatouk, I.; Bouaïcha, N.; Fontan, D.; Levi, Y. Seasonal variation of microcystin concentrations in the Saint-Caprais reservoir (France) and their removal in a small full-scale treatment plant. *Water Research* **2002**, *36*, doi:10.1016/S0043-1354(01)00507-3.
42. Furey, A.; Crowley, J.; Shuilleabhain, A.N.; Skulberg, O.M.; James, K.J. The first identification of the rare cyanobacterial toxin, homoanatoxin-a, in Ireland. *Toxicon* **2003**, *41*, doi:10.1016/S0041-0101(02)00291-X.
43. Carrasco, D.; Moreno, E.; Sanchis, D.; Wörmer, L.; Paniagua, T.; Cueto, A.D.; Quesada, A. Cyanobacterial abundance and microcystin occurrence in Mediterranean water reservoirs in Central Spain: microcystins in the Madrid area. *European Journal of Phycology* **2006**, doi:10.1080/09670260600801724.
44. Barco, M.; Flores, C.; Rivera, J.; Caixach, J. Determination of microcystin variants and related peptides present in a water bloom of Planktothrix (Oscillatoria) rubescens in a Spanish drinking water reservoir by LC/ESI-MS. *Toxicon* **2004**, *44*, doi:10.1016/j.toxicon.2004.08.011.
45. Cirés, S.; Wörmer, L.; Carrasco, D.; Quesada, A. Sedimentation Patterns of Toxin-Producing Microcystis Morphospecies in Freshwater Reservoirs. *Toxins* **2013**, *5*, 939-957, doi:10.3390/toxins5050939.
46. Wörmer, L.; Agha, R.; Cirés, S. *Informe de los análisis realizados en las zonas de baño continentales durante las temporadas 2008 y 2009*; 2011.
47. Lezcano, M.Á.; Morón-López, J.; Agha, R.; López-Heras, I.; Nozal, L.; Quesada, A.; El-Shehawy, R. Presence or Absence of mlr Genes and Nutrient Concentrations Co-Determine the Microcystin Biodegradation Efficiency of a Natural Bacterial Community. *Toxins* **2016**, *8*, doi:10.3390/toxins8110318.
48. Svirčev, Z.; Krstič, S.; Miladinov-Mikov, M.; Baltič, V.; Vidovič, M. Freshwater Cyanobacterial Blooms and Primary Liver Cancer Epidemiological Studies in Serbia. *Journal of Environmental Science and Health Part C* **2009**, *27*, 36–55, doi:10.1080/10590500802668016.
49. Stoyneva-Gärtner, M.; Stefanova, K.; Uzunov, B.; Radkova, M.; Gärtner, G.; Stoyneva-Gärtner, M.; Stefanova, K.; Uzunov, B.; Radkova, M.; Gärtner, G. Cuspidothrix Is the First Genetically Proved Anatoxin A Producer in Bulgarian Lakes and Reservoirs. *Toxins* **2022**, *14*, doi:10.3390/toxins14110778.
50. Teneva, I.; Mladenov, R.; Belkinova, D.; Dimitrova-Dyulgerova, I.; Dzhambazov, B. Phytoplankton community of the drinking water supply reservoir Borovitsa (South Bulgaria) with an emphasis on cyanotoxins and water quality. *Central European Journal of Biology* **2010**, *5*, doi:10.2478/s11535-010-0009-1.

51. Teneva, I.; Belkinova, D.; Dimitrova-Dyulgerova, I. Phytoplankton assemblages and monitoring of cyanotoxins in Trakiets Reservoir. In *Scientific Researches of the Union of Scientists in Bulgaria - Plovdiv, series B. Natural Sciences and the Humanities*; 2009; pp. 244–249.
52. Pavlova, V.; Stoyneva, M.; Georgieva, V.; Donchev, D.; Spoof, L.; Meriluoto, J.; Bratanova, Z.; Karadjova, I. New Records of Microcystins in Some Bulgarian Water Bodies of Health and Conservational Importance. *Journal of Water Resource and Protection* **2014**, *6*, doi:10.4236/jwarp.2014.65044.
53. Pavlova, V.; Stoyneva-Gärtner, M.; Uzunov, B. Microcystins-LR, -YR and -RR in six Bulgarian water bodies of health and conservational importance (2012-2014). *Water Resource and Protection* **2015**, *7*, 1375-1386.
54. Georgieva, V.; Pavlova, V.; Pchelina, Z. Hygienic assessment of the water reservoirs "Studena", "Bistritsa" and "Pchelina", based on performed hydrobiological analysis and determination of some toxins. *Bulgarian Journal of Public Health* **2015**, *7*, 3-13.
55. Kaloudis, T.; Zervou, S.-K.; Tsimeli, K.; Triantis, T.M.; Fotiou, T.; Hiskia, A. Determination of microcystins and nodularin (cyanobacterial toxins) in water by LC–MS/MS. Monitoring of Lake Marathonas, a water reservoir of Athens, Greece. *Journal of Hazardous Materials* **2013**, *263*, doi:10.1016/j.jhazmat.2013.07.036.
56. Jančula, D.; Straková, L.; Sadílek, J.; Maršálek, B.; Babica, P. Survey of cyanobacterial toxins in Czech water reservoirs—the first observation of neurotoxic saxitoxins. *Environmental Science and Pollution Research* **2014**, *21*, 8006–8015, doi:10.1007/s11356-014-2699-9.
57. Mooney, K.M.; Hamilton, J.T.G.; Floyd, S.D.; Foy, R.H.; Elliott, C.T. Initial studies on the occurrence of cyanobacteria and microcystins in Irish lakes. *Environmental Toxicology* **2011**, *26*, 566–570, doi:10.1002/tox.20577.
58. Schmidt, W.; Willmitzer, H.; Bornmann, K.; Pietsch, J. Production of drinking water from raw water containing cyanobacteria—pilot plant studies for assessing the risk of microcystin breakthrough. *Environmental Toxicology* **2002**, *17*, doi:10.1002/tox.10067.
59. Messineo, V.; Bogialli, S.; Melchiorre, S.; Sechi, N.; Lugliè, A.; Casiddu, P.; Mariani, M.A.; Padedda, B.M.; Corcia, A.D.; Mazza, R.; et al. Cyanobacterial toxins in Italian freshwaters. *Limnologica* **2009**, *39*, doi:10.1016/j.limno.2008.09.001.
60. Gregorio, F.N.D.; Bogialli, S.; Ferretti, E.; Lucentini, L. First evidence of MC-HtyR associated to a Plankthothrix rubescens blooming in an Italian lake based on a LC-MS method for routinely analysis of twelve microcystins in freshwaters. *Microchemical Journal* **2017**, *130*, doi:10.1016/j.microc.2016.10.012.
61. Stefanelli, M.; Scardala, S.; Cabras, P.A.; Orrù, A.; Vichi, S.; Testai, E.; Funari, E.; Manganelli, M. Cyanobacterial dynamics and toxins concentrations in Lake Alto Flumendosa, Sardinia, Italy. *Advances in Oceanography and Limnology* **2017**, *8*, 71–86, doi:10.4081/aiol.2017.6352.
62. Pekar, H.; Westerberg, E.; Bruno, O.; Lääne, A.; Persson, K.M.; Sundström, L.F.; Thim, A.-M. Fast, rugged and sensitive ultra high pressure liquid chromatography tandem mass spectrometry method for analysis of cyanotoxins in raw water and drinking water—First findings of anatoxins, cylindrospermopsins and microcystin variants in Swedish source waters and infiltration ponds. *Journal of Chromatography A* **2016**, *1429*, 265–276, doi:10.1016/j.chroma.2015.12.049.
63. Chernova, E.; Sidelev, S.; Russkikh, I.; Voyakina, E.; Babanazarova, O.; Romanov, R.; Kotovshchikov, A.; Mazur-Marzec, H. Dolichospermum and Aphanizomenon as neurotoxins producers in some Russian freshwaters. *Toxicon* **2017**, *130*, doi:10.1016/j.toxicon.2017.02.016.
64. Chernova, E.; Sidelev, S.; Russkikh, I.; Korneva, L.; Solovyova, V.; Mineeva, N.; Stepanova, I.; Zhakovskaya, Z. Spatial distribution of cyanotoxins and ratios of microcystin to biomass indicators in the reservoirs of the Volga, Kama and Don Rivers, the European part of Russia. *Limnologica* **2020**, *84*, 125819, doi:10.1016/j.limno.2020.125819.
65. Azevedo, S.M.F.O.; Carmichael, W.W.; Jochimsen, E.M.; Rinehart, K.L.; Lau, S.; Shaw, G.R.; Eaglesham, G.K. Human intoxication by microcystins during renal dialysis treatment in Caruaru—Brazil. *Toxicology* **2002**, *181-182*, 441–446, doi:10.1016/S0300-483X(02)00491-2.

66. Vieira, J.M.d.S.; Azevedo, M.T.d.P.; Azevedo, S.M.F.d.O.; Honda, R.Y.; Corrêa, B. Microcystin production by Radiocystis fernandoi (Chroococcales, Cyanobacteria) isolated from a drinking water reservoir in the city of Belém, PA, Brazilian Amazonia region. *Toxicon* **2003**, *42*, doi:10.1016/j.toxicon.2003.08.004.
67. Vieira, J.M.d.S.; Azevedo, M.T.d.P.; Azevedo, S.M.F.d.O.; Honda, R.Y.; Corrêa, B. Toxic cyanobacteria and microcystin concentrations in a public water supply reservoir in the Brazilian Amazonia region. *Toxicon* **2005**, *45*, doi:10.1016/j.toxicon.2005.02.008.
68. Becker, V.; Ihara, P.; Yunes, J.S.; Huszar, V.L.M. Occurrence of anatoxin-a(s) during a bloom of Anabaena crassa in a water-supply reservoir in southern Brazil. *Journal of Applied Phycology* **2009**, *22*, doi:10.1007/s10811-009-9451-8.
69. Lorenzi, A.S.; Chia, M.A.; Lopes, F.A.C.; Silva, G.G.Z.; Edwards, R.A.; Bittencourt-Oliveira, M.d.C. Cyanobacterial biodiversity of semiarid public drinking water supply reservoirs assessed via next-generation DNA sequencing technology. *Journal of Microbiology* **2019**, *57*, doi:10.1007/s12275-019-8349-7.
70. Lins, R.P.M.; Barbosa, L.G.; Minillo, A.; De Ceballos, B.S.O. Cyanobacteria in a eutrophicated reservoir in a semi-arid region in Brazil: dominance and microcystin events of blooms. *Brazilian Journal of Botany* **2016**, *39*, doi:10.1007/s40415-016-0267-x.
71. Calado, S.L.d.M.; Santos, G.S.; Wojciechowski, J.; Magalhães, V.F.d.; Assis, H.C.S.d. The accumulation dynamics, elimination and risk assessment of paralytic shellfish toxins in fish from a water supply reservoir. *Science of The Total Environment* **2019**, *651*, doi:10.1016/j.scitotenv.2018.10.046.
72. Oliveira, E.D.C.; Castelo-Branco, R.; Silva, L.; Silva, N.; Azevedo, J.; Vasconcelos, V.; Faustino, S.; Cunha, A. First Detection of Microcystin-LR in the Amazon River at the Drinking Water Treatment Plant of the Municipality of Macapá, Brazil. *Toxins* **2019**, *11*, 669, doi:10.3390/toxins11110669.
73. Passos, L.S.; Almeida, É.C.d.; Villela, A.; Fernandes, A.N.; Marinho, M.M.; Gomes, L.C.; Pinto, E. Cyanotoxins and water quality parameters as risk assessment indicators for aquatic life in reservoirs. *Ecotoxicology and Environmental Safety* **2022**, *241*, doi:10.1016/j.ecoenv.2022.113828.
74. Moraes, M.A.B.; Rodrigues, R.A.M.; Schlüter, L.; Podduturi, R.; Jørgensen, N.O.G.; Calijuri, M.C.; Moraes, M.A.B.; Rodrigues, R.A.M.; Schlüter, L.; Podduturi, R.; et al. Influence of Environmental Factors on Occurrence of Cyanobacteria and Abundance of Saxitoxin-Producing Cyanobacteria in a Subtropical Drinking Water Reservoir in Brazil. *Water* **2021**, *13*, doi:10.3390/w13121716.
75. Bertrand, L.; Iturburu, F.; Valdés, M.; Menone, M.; Amé, M. Risk evaluation and prioritization of contaminants of emerging concern and other organic micropollutants in two river basins of central Argentina. *The Science of the total environment* **2023**, *878*, doi:10.1016/j.scitotenv.2023.163029.
76. Conti, A.L.R.; Guerrero, J.M.; Regueira, J.M. Levels of microcystins in two argentinean reservoirs used for water supply and recreation: Differences in the implementation of safe levels. *Environmental Toxicology* **2005**, *20*, doi:10.1002/tox.20107.
77. Ruiz, M.; Galanti, L.; Ruibal, A.L.; Rodriguez, M.I.; Wunderlin, D.A.; Amé, M.V. First Report of Microcystins and Anatoxin-a Co-occurrence in San Roque Reservoir (Córdoba, Argentina). *Water, Air, & Soil Pollution* **2013**, *224*, 1–17, doi:10.1007/s11270-013-1593-2.
78. Giannuzzi, L.; Carvajal, G.; Corradini, M.; Araujo, A.C.; Echenique, R.; Andrinolo, D. Occurrence of toxic cyanobacterial blooms in rio de la plata estuary, Argentina: field study and data analysis - PubMed. *Journal of toxicology* **2012**, *2012*, doi:10.1155/2012/373618.
79. Forastier, M.; Zalocar, Y.; Andrinolo, D.; Domitrovic, H.A. Occurrence and toxicity of Microcystis aeruginosa (Cyanobacteria) in the Paraná River, downstream of the Yacyretá dam (Argentina). *Revista de Biología Tropical* **2016**, *64*, 203-211, doi:http://dx.doi.org/10.15517/rbt.v64i1.8993.
80. Almanza, V.; Parra, O.; De M. Bicudo, C.E.; Baeza, C.; Beltran, J.; Figueroa, R.; Urrutia, R. Occurrence of toxic blooms of Microcystis aeruginosa in a central Chilean (36° Lat. S) urban lake. *Revista Chilena de Historia Natural* **2016**, *89*, doi:10.1186/s40693-016-0057-7.

81. León, C.; Peñuela, G.A. Detected cyanotoxins by UHPLC MS/MS technique in tropical reservoirs of northeastern Colombia. *Toxicon* **2019**, *167*, doi:10.1016/j.toxicon.2019.06.010.
82. Munoz, M.; Cirés, S.; Pedro, Z.M.d.; Colina, J.Á.; Velásquez-Figueroa, Y.; Carmona-Jiménez, J.; Caro-Borrero, A.; Salazar, A.; Fuster, M.-C.S.M.; Contreras, D.; et al. Overview of toxic cyanobacteria and cyanotoxins in Ibero-American freshwaters: Challenges for risk management and opportunities for removal by advanced technologies. *Science of The Total Environment* **2021**, *761*, doi:10.1016/j.scitotenv.2020.143197.
83. Kotak, B.G.; Lam, A.K.-Y.; Prepas, E.E.; Kenefick, S.L.; Hrudey, S.E. Variability of the hepatotoxin, microcystin-LR, in hypereutrophic drinking water lakes. *Journal of Phycology* **1995**, *31*, doi:10.1111/j.0022-3646.1995.00248.x.
84. Chen, H.; Burke, J.M.; Dinsmore, W.P.; Prepas, E.E.; Fedorak, P.M. First assessment of cyanobacterial blooms and microcystin-LR in the Canadian portion of Lake of the Woods. *Lake and Reservoir Management* **2007**, *23*, doi:10.1080/07438140709353920.
85. Izaguirre, G.; Jungblut, A.-D.; Neilan, B.A. Benthic cyanobacteria (Oscillatoriaceae) that produce microcystin-LR, isolated from four reservoirs in southern California. *Water Research* **2007**, *41*, doi:10.1016/j.watres.2006.10.012.
86. Steffen, M.M.; Davis, T.W.; McKay, R.M.L.; Bullerjahn, G.S.; Krausfeldt, L.E.; Stough, J.M.A.; Neitzey, M.L.; Gilbert, N.E.; Boyer, G.L.; Johengen, T.H.; et al. Ecophysiological Examination of the Lake Erie Microcystis Bloom in 2014: Linkages between Biology and the Water Supply Shutdown of Toledo, OH. *Environmental Science & Technology* **2017**, *51*, 6745–6755, doi:10.1021/acs.est.7b00856.
87. Ai, Y.; Lee, S.; Lee, J. Drinking water treatment residuals from cyanobacteria bloom-affected areas: Investigation of potential impact on agricultural land application. *Science of The Total Environment* **2020**, *706*, doi:10.1016/j.scitotenv.2019.135756.
88. Bolotaolo, M.; Kurobe, T.; Puschner, B.; Hammock, B.G.; Hengel, M.J.; Lesmeister, S.; Teh, S.J. Analysis of Covalently Bound Microcystins in Sediments and Clam Tissue in the Sacramento–San Joaquin River Delta, California, USA. *Toxins 2020, Vol. 12, Page 178* **2020**, *12*, doi:10.3390/toxins12030178.
89. Melaram, R.; Lopez-Dueñas, B. Frontiers | Detection and Occurrence of Microcystins and Nodularins in Lake Manatee and Lake Washington-Two Floridian Drinking Water Systems. *Frontiers in Water* **2022**, *4*, doi:10.3389/frwa.2022.899572.
90. Mercado Borrayo, B.M. *Estudio sobre la remoción de cianobacterias y sus metabolitos en la Planta Potabilizadora "Los Berros" Sistema Cutzamala*; Universidad Nacional Autónoma de México: 2007.
91. Nandini, S.; Sánchez-Zamora, C.; Sarma, S.S.S. Toxicity of cyanobacterial blooms from the reservoir Valle de Bravo (Mexico): A case study on the rotifer Brachionus calyciflorus. *Science of The Total Environment* **2019**, *688*, doi:10.1016/j.scitotenv.2019.06.297.
92. Fernández, R.; Alcocer, J.; Oseguera, L.A. Microcystins presence threatens the ecosystem health of a tropical National Park: Lagunas de Montebello, Chiapas. *Brazilian Journal of Botany* **2021**, *44*, doi:10.1007/s40415-020-00686-5.
93. Martínez-Jerónimo, F.; Antuna-González, P.d.C.; Hernández-Zamora, M.; Martínez-Jerónimo, L.; Munoz, G.; Simon, D.F.; Sauvé, S. Year-long monitoring of phytoplankton community, toxigenic cyanobacteria, and total microcystins in a eutrophic tropical dam supplying the Mexico megacity. *Frontiers in Environmental Science* **2022**, *10*, 984365, doi:10.3389/fenvs.2022.984365.
